# Integrative analysis of the 3D genome and epigenome in mouse embryonic tissues

**DOI:** 10.1038/s41594-024-01431-2

**Published:** 2024-12-16

**Authors:** Miao Yu, Nathan R. Zemke, Ziyin Chen, Ivan Juric, Rong Hu, Ramya Raviram, Armen Abnousi, Rongxin Fang, Yanxiao Zhang, David U. Gorkin, Yang E. Li, Yuan Zhao, Lindsay Lee, Shreya Mishra, Anthony D. Schmitt, Yunjiang Qiu, Diane E. Dickel, Axel Visel, Len A. Pennacchio, Ming Hu, Bing Ren

**Affiliations:** 1 State Key Laboratory of Genetic Engineering, School of Life Sciences, Fudan University, Shanghai, China; 2 Ludwig Institute for Cancer Research, La Jolla, CA USA; 3 Department of Cellular and Molecular Medicine, University of California, San Diego School of Medicine, La Jolla, CA USA; 4 Center for Epigenomics, University of California, San Diego School of Medicine, La Jolla, CA USA; 5 Department of Quantitative Health Sciences, Lerner Research Institute, Cleveland Clinic Foundation, Cleveland, OH USA; 6 Center for Immunology and Precision Immuno-Oncology, Lerner Research Institute, Cleveland Clinic Foundation, Cleveland, OH USA; 7 Bioinformatics and Systems Biology Graduate Program, University of California, San Diego, La Jolla, CA USA; 8 UCSD Biomedical Sciences Graduate Program, University of California, San Diego, La Jolla, CA USA; 9 Environmental Genomics and Systems Biology Division, Lawrence Berkeley National Laboratory, Berkeley, CA USA; 10 US Department of Energy Joint Genome Institute, Berkeley, CA USA; 11 School of Natural Sciences, University of California, Merced, Merced, CA USA; 12 Comparative Biochemistry Program, University of California, Berkeley, Berkeley, CA USA; 13 Present Address: New York Genome Center, New York, NY USA; 14 Present Address: Meta, Bellevue, WA USA; 15 Present Address: Howard Hughes Medical Institute, Department of Chemistry and Chemical Biology, Harvard University, Cambridge, MA USA; 16 Present Address: School of Life Sciences, Westlake University, Hangzhou, China; 17 Present Address: Department of Biology, Emory University, Atlanta, GA USA; 18 Present Address: Department of Neurosurgery and Genetics, Washington University School of Medicine, St. Louis, MO USA; 19 Present Address: Arima Genomics, Inc., San Diego, CA USA; 20 Present Address: Sana Biotechnology, Seattle, WA USA

**Keywords:** Epigenomics, Developmental biology, Transcriptional regulatory elements, Chromatin structure

## Abstract

While a rich set of putative *cis*-regulatory sequences involved in mouse fetal development have been annotated recently on the basis of chromatin accessibility and histone modification patterns, delineating their role in developmentally regulated gene expression continues to be challenging. To fill this gap, here we mapped chromatin contacts between gene promoters and distal sequences across the genome in seven mouse fetal tissues and across six developmental stages of the forebrain. We identified 248,620 long-range chromatin interactions centered at 14,138 protein-coding genes and characterized their tissue-to-tissue variations and developmental dynamics. Integrative analysis of the interactome with previous epigenome and transcriptome datasets from the same tissues revealed a strong correlation between the chromatin contacts and chromatin state at distal enhancers, as well as gene expression patterns at predicted target genes. We predicted target genes of 15,098 candidate enhancers and used them to annotate target genes of homologous candidate enhancers in the human genome that harbor risk variants of human diseases. We present evidence that schizophrenia and other adult disease risk variants are frequently found in fetal enhancers, providing support for the hypothesis of fetal origins of adult diseases.

## Abstract (web summary)

The authors here show that chromatin interactions during mouse fetal development are spatiotemporally dynamic. Integrating interactomes with other datasets predicts target genes for candidate enhancers and helps interpret noncoding risk variants in the human genome.

## Main

Developmental programs in multicellular organisms involve temporal and spatially controlled expression of genes, which are driven by the dynamic binding of sequence-specific transcription factors (TFs) to *cis*-regulatory elements (CREs)^1–5^. Recently, the Encyclopedia of DNA Elements (ENCODE)^6,7^ project annotated a rich set of candidate CREs (cCREs) in the mouse genome across a wide spectrum of tissues and early developmental stages^8,9^. While these catalogs of cCREs have revealed the location and tissue-specific usage of regulatory sequences in the genome, understanding their role in developmental programs remains a daunting challenge because of our incomplete knowledge of the regulatory target genes of most cCREs.

Active cCREs (for example, enhancers) and their target gene promoters are believed to be spatially close in three-dimensional (3D) space. Consequently, high-throughput chromatin conformation capture-based techniques, such as Hi-C^10,11^, capture Hi-C^12,13^, ChIA-PET^14,15^, HiChIP^16^ and PLAC-seq^17^, have been increasingly used to map long-range chromatin interactions between promoters and cCREs to infer their target genes across a wide spectrum of human and mouse tissues (or cell types)^18–23^. It has been demonstrated that tissue-specific interactions are not only correlated with changes in gene expression but also enriched for tissue-specific cCREs and noncoding risk variants associated with diseases, revealing the power of using 3D genome information to identify the function of cCREs and their contribution to disease etiology. However, the majority of previous studies focused on adult tissues and the chromatin interactomes in the developing fetus have yet to be systematically investigated.

In this work, we mapped chromatin interactions centered at 14,138 protein-coding gene promoters in mouse embryos, spanning seven tissues and, for one of them, across six developmental stages. To investigate the role of CCCTC-binding factor (CTCF) in mediating the promoter-centered interactions, we also generated CTCF ChIP-seq datasets using the same samples. Integrating with epigenomic profiles (eight histone modifications, chromatin accessibility)^8^ and gene expression landscapes^24^ from the same tissues and developmental stages, we examined the relationships among long-range chromatin interactions, chromatin states, chromatin accessibility, CTCF binding and gene expression during mouse fetal development. We predicted enhancer target genes and explained tissue-specific and developmental stage-restricted gene expression programs. We further demonstrated that 3D genome information may facilitate the functional annotation of noncoding risk variants in the human genome and prioritization of gene targets for complex human diseases.

## Results

### Promoter-centered chromatin interactome in mouse fetal tissues

**Fig. 1:**
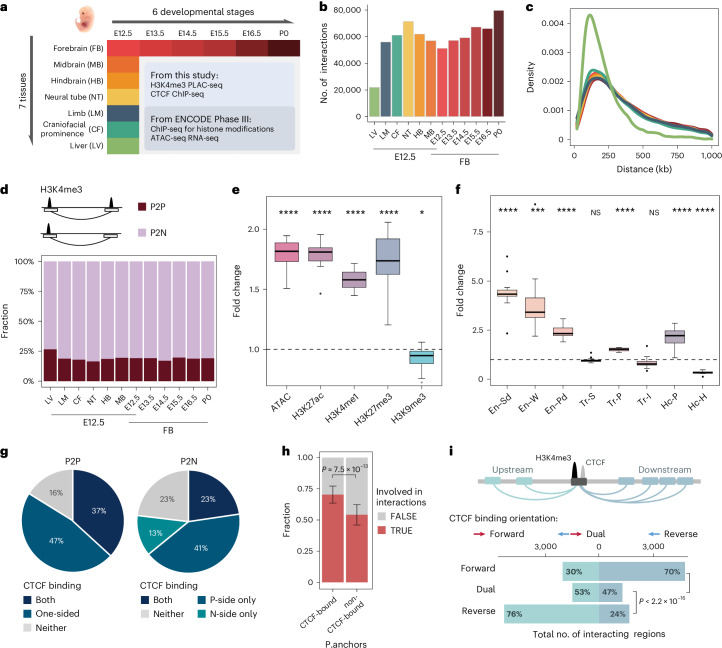
Characteristics of promoter-centered interactions identified from H3K4me3 PLAC-seq across 12 tissues and developmental stages during mouse fetal development. **a**, Overview of the experimental design. **b**, Number of MAPS-identified chromatin interactions. **c**, The density plot of interaction distance across 12 tissues, each represented by distinct colors shown in **b**. **d**, Fraction of P2P and P2N interactions across 12 samples. **e**,**f**, Box plots showing the enrichment of promoter-interacting regions for accessible chromatins: histone marks of P2N interactions (**e**) and different chromatin states (**f**). A fold change of 1 is marked by the horizontal dashed line (*n* = 12). En–Sd, strong TSS–distal enhancer; En–W, weak TSS–distal enhancer; En–Pd, poised TSS–distal enhancer; Tr-S, strong transcription; Tr-P, permissive transcription; Tr-I, initiation transcription; Hc-P, Polycomb-associated heterochromatin; Hc-H, H3K9me3-associated heterochromatin. Central bar, median; lower and upper box limits, 25th and 75th percentiles, respectively; whiskers, minimum and maximum value within the range of (first quartile − 1.5 × (third quartile − first quartile)) to (third quartile + 1.5 × (third quartile − first quartile)). Two-tailed single sample *t*-test comparing to *μ* = 1, with FDR multiple comparison adjustment. *P ≤ 0.05, ***P ≤ 0.001 and ****P ≤ 0.0001. **g**, Average proportion of long-range interactions classified according to presence or absence of CTCF binding on one or both ends across 12 tissues (*n* = 12). A P2P interaction might be bound by CTCF at both ends (both), one of the two ends (one-sided) or neither end (neither). A P2N interaction might be bound by CTCF at both ends (both), only the promoter end (P-side only), only the nonpromoter end (N-side only) or neither end (neither). **h**, Average fraction of candidate interaction anchors involved in long-range interactions (*n* = 12). Anchors bound or not bound by CTCF are considered separately. Data are presented as the mean values ± s.d. Two-tailed paired *t*-test. **i**, A bar plot showing the number and the fraction of upstream (light cyan) and downstream (light blue) distal-interacting regions that form interactions with the promoter anchors bound by CTCF in forward, reverse or dual orientation in FB E12.5. *P* values were calculated using the chi-square test.

We performed PLAC-seq experiments (also known as HiChIP) using antibodies against histone H3 lysine 4 trimethylation (H3K4me3), a chromatin mark found at both active and poised promoter regions, to map chromatin interactions centered at gene promoters. Seven mouse fetal tissues from embryonic day 12.5 (E12.5) were assayed in duplicates, including the forebrain (FB), midbrain (MB), hindbrain (HB), neural tube (NT), limb (LM), craniofacial prominence (CF) and liver (LV). FB tissues at five extra time points from E13.5 until birth (E13.5, E14.5, E15.5, E16.5 and postnatal day 0 (P0)) were further investigated (Fig. 1a). Two biological replicates were collected and assayed for each tissue, with a median sequencing depth of 206 million read pairs per replicate. Each replicate was subjected to a set of key quality control criteria to ensure the consistent data quality across different samples (Supplementary Table 1 and Methods).

**Extended Data Fig. 1:**
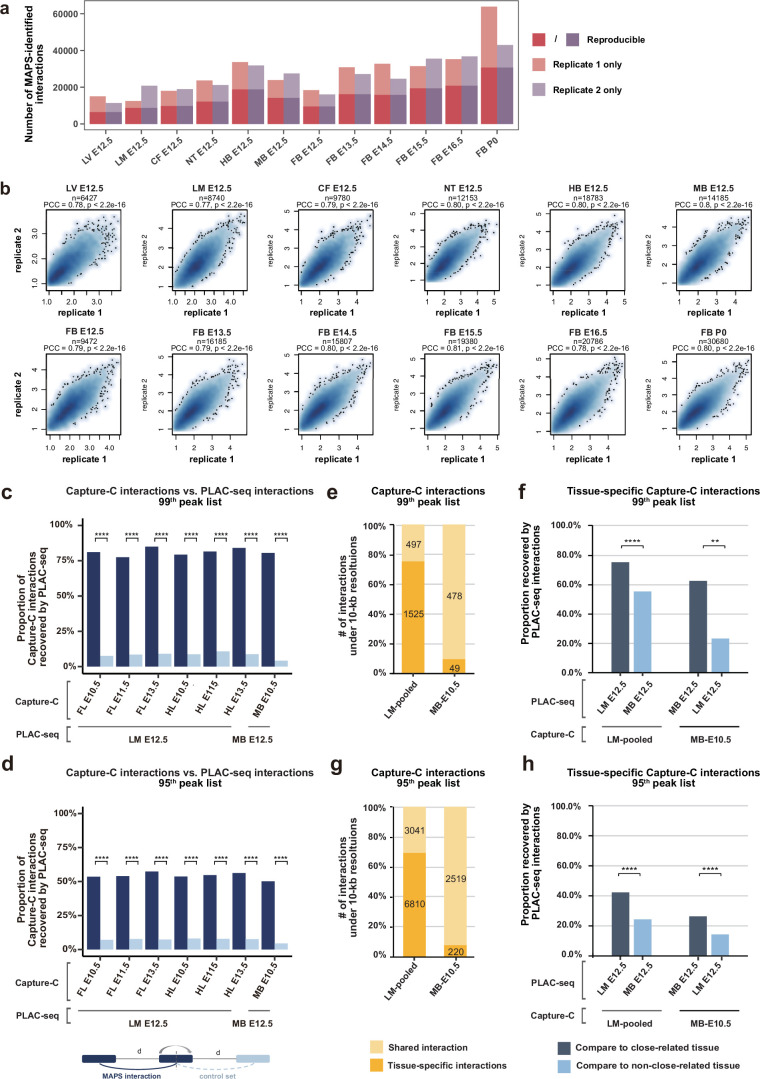
Benchmark the quality of H3K4me3 PLAC-seq data. **a**. A bar-plot showing the number of MAPS-identified interactions from each biological replicate with proportions of reproducible interactions highlighted by darker colors. **b**. Scatterplots showing the reproducibility of the normalized contact frequencies between two biological replicates across 12 tissue and stages. PCC: Pearson correlation coefficients. P-values from Pearson correlation test. **c**, **d**. Proportions of interactions identified from published Capture-C datasets^26^ using 99^th^ (**c**) or 95^th^ (**d**) percentile as thresholds that overlap with those identified from H3K4me3 PLAC-seq data using the closest tissues (See Methods). Pseudo-interactions that link H3K4me3-containing bin with bin equidistant from but on the other side of H3K4me3-containing bins were used as the control set. FL, forelimb. HL, hindlimb. MB, midbrain. E, embryonic day. **e**, **g**. Percent of interactions identified from published Capture-C datasets^26^ specifically existed in one tissue type or shared by both types of tissues under 10-kb resolution, using 99^th^ (**e**) or 95^th^ (**g**) percentile of the empirical distribution as thresholds. **f**, **h**. Proportions of tissue-specific Capture-C interactions (the orange part in **e**, **g**) that overlap with those identified from H3K4me3 PLAC-seq data from midbrain E12.5 or limb E12.5 from PLAC-seq. Comparison between closely-related tissues (limb vs. limb, midbrain vs midbrain) are colored in dark blue while comparison between non-closely-related tissues (limb vs. midbrain) are colored in light blue. **** Chi-square P-value < 2.2e-16.

To check the reproducibility between biological replicates, we first used MAPS^25^ to identify statistically significant long-range chromatin interactions from each biological replicate at 10-kb resolution. We found 54–71% of interactions were shared between two biological replicates (Extended Data Fig. 1a), which is at a similar level to our previous study using mouse embryonic stem cells^25^. Additionally, the normalized contact frequencies at the reproducible interactions were also highly correlated, with Pearson correlation coefficients (PCCs) ranging 0.77–0.80 (Extended Data Fig. 1b). We, therefore, combined the data from two replicates of each tissue to increase the sensitivity of chromatin interaction detection. The combined data were further downsampled so that all 12 tissue–time point combinations had the same number of usable reads. After reapplying MAPS to these sample-balanced datasets, a total of 248,620 interactions were identified at 10-kb resolution across all tissues within a genomic distance of 20 kb to 1 Mb. A total of 51,264–79,677 interactions were identified in each tissue excluding LV E12.5, where only 21,945 interactions were found (Fig. 1b). The median genomic distances that these interactions spanned were 270–310 kb for 11 of the tissues, whereas, in LV E12.5, the median distance was substantially shorter (170 kb) (Fig. 1c). Overall, 16–27% (median: 19%) of the identified interactions were between two H3K4me3-marked regions (hereinafter referred to as ‘promoter-to-promoter’ (P2P) interactions), whereas the remaining interactions (73–84%, median: 81%) were between a genomic region associated with H3K4me3 and another one displaying no H3K4me3 signals (hereinafter referred to as ‘promoter-to-nonpromoter’ (P2N) interactions) (Fig. 1d).

To evaluate the sensitivity of H3K4me3 PLAC-seq in detecting promoter-centered interactions, we compared our MAPS-identified interaction lists with published ones centered on 446 gene loci during mouse LM development revealed by Capture-C^26^ and found that the majority of Capture-C interactions were recapitulated by H3K4me3 PLAC-seq experiments from the most closely related tissues (Extended Data Fig. 1c,d). In addition, we observed a significantly higher recovery rate when comparing tissue-specific Capture-C interactions with PLAC-seq data of closely related tissues than those from nonclosely related tissues (Extended Data Fig. 1e–h).

### P2N interactions frequently occur between putative enhancers and promoters

**Extended Data Fig. 2:**
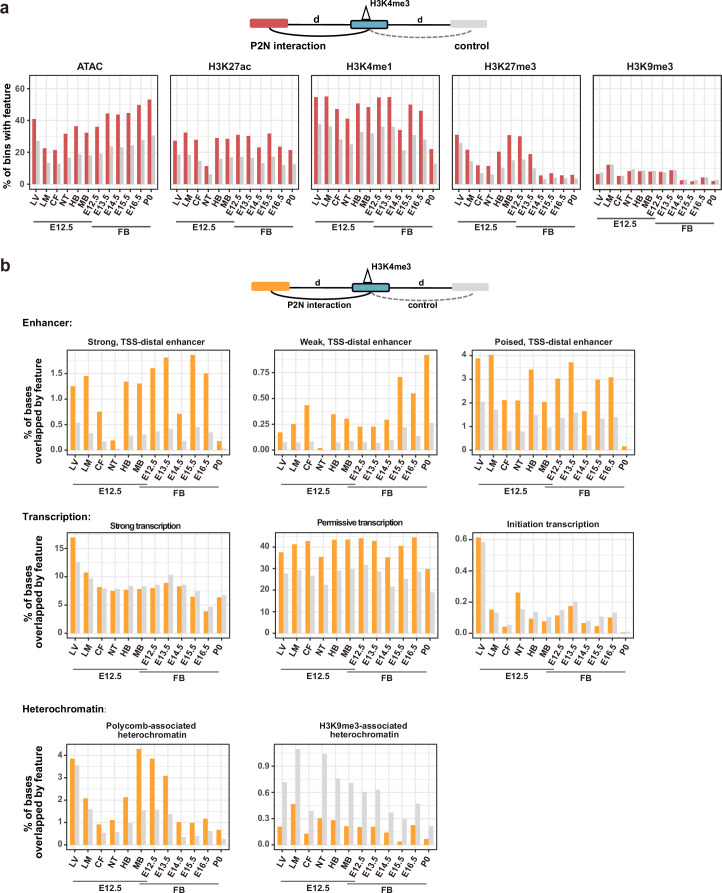
Promoter-interacting regions are significantly enriched for accessible chromatin and specific histone marks across all 12 tissues. **a**. Bar plots showing the percent of promoter-interacting bins overlapping the reproducible peaks identified from ATAC-seq, H3K27ac ChIP-seq, H3K4me1 ChIP-seq, H3K27me3 ChIP-seq or H3K9me3 ChIP-seq from the same tissue. Bins with equidistant from but on the other side of H3K4me3-containing bins were used as the control set, as shown in the schematic diagram on the top. **b**. Bar plots showing the fraction of base pairs in promoter-interacting bins overlapping the reproducible chromHMM state calls from the same tissue, categorized by enhancer (top), transcription (middle), and heterochromatin (bottom) status. Bins with equidistant from but on the other side of H3K4me3-containing bins were used as the control set, as shown in the schematic diagram on the top.

As demonstrated in Fig. 1d, >80% of the identified interactions were P2N interactions, indicating that these interactions may link promoters to their potential regulatory elements. Indeed, across all 12 tissues, on average, 38% of those promoter-interacting regions in P2N interactions contained accessible chromatin regions, defined by ATAC-seq peaks^8^ and such a proportion (that is, 38%) was significantly (*P* = 1.89 × 10^−10^, one-sample *t*-test comparing fold change to *μ* = 1) higher than the distance-matched control regions (21%) (Fig. 1e and Extended Data Fig. 2a). Further exploration of their chromatin properties using ChIP-seq data of histone modifications^8^ suggested that they were likely enhancers; both H3K4me1 (47% in P2N versus 30% in control, *P* = 5.62 × 10^−11^) and H3K27ac (26% in P2N versus 15% in control, *P* = 2.41 × 10^−10^) were enriched at these regions. The occurrence of H3K27me3 at these regions was also significantly higher than that in control regions (17% versus 10%, *P* = 4.42 × 10^−7^), consistent with previous reports of Polycomb-associated chromatin interactions^27–29^. In contrast, the heterochromatin mark H3K9me3 was depleted (6% versus 7%, *P* = 0.015) (Fig. 1e and Extended Data Fig. 2a). In line with above observations, integrative analysis with the 15-state ChromHMM model^30^ revealed 2–9-fold enrichment of distal enhancers (strong, weak and poised) and depletion of H3K9me3-associated heterochromatins at these nonpromoter regions (Fig. 1f and Extended Data Fig. 2b). Taken together, our data suggest that promoter-centered chromatin interactions frequently occur between promoters and putative enhancers in mouse fetal tissues, which is in line with previous findings in many other cell types and tissues^11,19,31^.

### CTCF is broadly involved in promoter-centered interactions during fetal development

**Extended Data Fig. 3:**
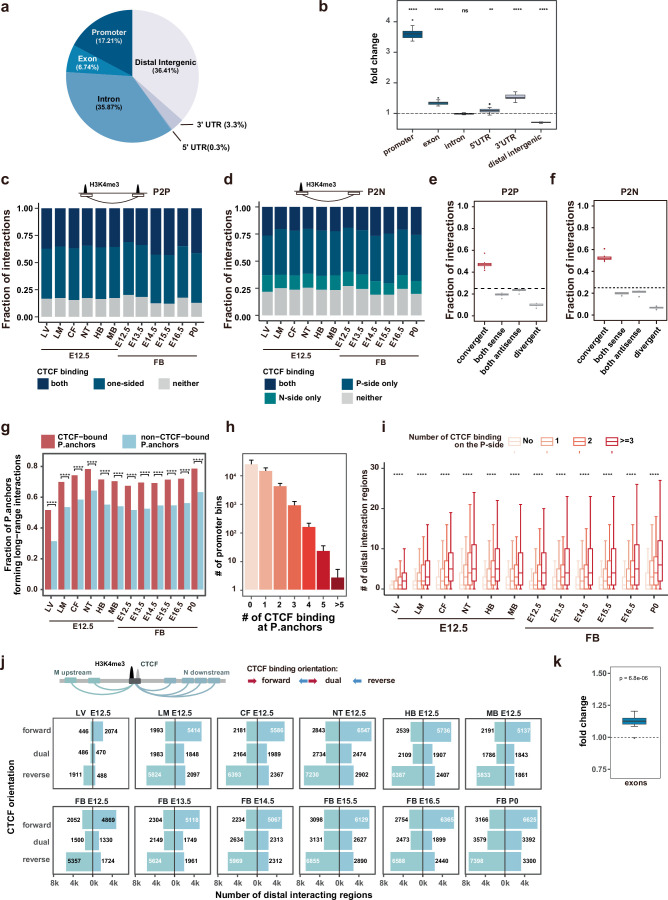
The role of CTCF in promoter-centered chromatin interactions. **a**. Pie chart for percentage of CTCF peaks averaged across tissues falling within indicated genomic annotation. **b**. Boxplots showing enrichment of replicated CTCF peaks inside indicated genomic annotation. Shuffled regions across genome served as control set. Dashed line marks a fold-change of 1 (n = 12). Two-tailed single sample t-test comparing to mu = 1 with FDR multiple comparison adjustment, ‘ns’: not significant, *p <= 0.05, ***p <= 0.001, ****p <= 0.0001, **c**, **d**. Proportion of P2P (**c**) or P2N (**d**) interactions classified according to presence or absence of CTCF binding on one or both ends in each tissue or developmental stage. **e**, **f**. CTCF motif orientation on P2P (**e**) and P2N (**f**) interactions with both ends containing CTCF binding. (n = 12). P-value < 2 × 10^−16^ from chi-square test in all 12 tissues by comparing to expected fraction 0.25 for all 4 orientation combinations. **g**. Fraction of H3K4me3-containing 10-kb bins bound or not bound by CTCF that involved in long-range interactions in 12 tissues. Data are mean values ± SD. Chi-square test, ****p <= 0.0001. **h**. Bar plot showing number of CTCF peaks in candidate promoter anchor bins in 12 tissue/stage (N = 12). Data are mean values ± SD. **i**. Boxplots showing number of distal regions interacting with candidate promoter anchor bins with different numbers of CTCF peaks. ****p < 2 × 10^−16^ from ANOVA test in all 12 tissues. **j**. Bar plots showing number of upstream (light cyan) and downstream (light blue) distal-interacting regions that interact with promoter anchors bound by CTCF in forward, reverse or dual orientation in 12 tissues. Chi-square test. **k**. Boxplot showing enrichment of exons at CTCF peaks located at non-promoter sides of P2N interactions. Shuffled CTCF peaks limiting inside all non-promoter 10-kb bins of P2N interactions were used as control. Dashed line shows fold-change 1 (n = 12). Two tailed one-sample t-test comparing to mu = 1 (n = 12). For all boxplots: Central bar, median; lower and upper box limits, 25th and 75th percentiles, respectively; whiskers, minimum and maximum value within the range of (first quartile – 1.5× (third quartile – first quartile)) to (third quartile + 1.5 × (third quartile – first quartile)).

CTCF has a critical role in the formation of topologically associating domains and CTCF–CTCF loops through a cohesin-driven loop extrusion process^32^. While many previous studies revealed the crucial role of CTCF in the embryonic developmental program by either creating conditional knockout mice or focusing on specific CTCF-binding sites^33^, it remains unclear to what extent CTCF is involved in chromatin organization globally during fetal development. To define genome-wide CTCF-binding sites, we performed ChIP-seq assays for CTCF in all 12 mouse fetal tissues and identified 43,838–62,782 high-quality peaks in each tissue (Supplementary Table 2). As previously reported^34,35^, the identified CTCF-binding sites were most enriched at promoter regions (Extended Data Fig. 3a,b; 17.2% versus 4.7% by chance, *P* = 6 × 10^−14^). Given such a feature, we expected that a large proportion of interaction-associated promoters may have CTCF binding nearby (that is, within the same 10-kb bin). Indeed, the promoter side of 68% promoter-centered chromatin interactions contained at least one CTCF peak (37% + 47% = 84% for P2P interactions and 23% + 41% = 64% for P2N interactions) (Fig. 1g). In addition, 37% of P2P interactions and 23% of P2N interactions had CTCF binding on both ends (Fig. 1g and Extended Data Fig. 3c,d). These CTCF–CTCF interactions showed a significant preference of convergent motif orientation, consistent with the loop extrusion model^32,36^ (Extended Data Fig. 3e,f).

To investigate whether promoter-proximal CTCF binding contributes to the formation of interactions, we asked whether the ability of promoters forming long-range interactions correlated with the presence of CTCF-binding sites nearby. We observed that, on average, 70% of CTCF-bound promoters formed long-range interactions, whereas only 54% of non-CTCF-bound promoters served as anchors of long-range interactions (fold change = 1.3, *P* = 7.3 × 10^−7^) (Fig. 1h and Extended Data Fig. 3g). Additionally, promoters with more CTCF-binding sites tended to have more interactions with distal regions (Extended Data Fig. 3h,i). Promoters bound by forward CTCF were more likely to interact with their downstream regions, whereas promoters bound by reverse CTCF were more likely to interact with their upstream regions (Fig. 1i and Extended Data Fig. 3j). Collectively, the above results demonstrate a potential role of promoter-proximal CTCF binding in promoting the formation of promoter-centered chromatin interactions during fetal development, a phenomenon that we previously observed in mouse embryonic stem cells^37^.

Apart from the involvement of CTCF binding at the promoter regions, we also observed the enrichment of CTCF binding at the nonpromoter sides of P2N interactions; 36% (versus 17.6% in control, *P* = 4.9 × 10^−13^, Student’s *t*-test comparing to *μ* = 1) of P2N interactions overlapped with CTCF peaks on their nonpromoter side. Joint analysis with the 15-state ChromHMM model showed that 53% of the 10-kb bins containing both enhancers and CTCF-binding sites interacted with at least one promoter. In contrast, for those 10-kb bins containing enhancers but no CTCF-binding sites, only 32% of them formed interactions (fold change = 1.7, *P* = 2.5 × 10^−8^), indicating that the promoter-distal CTCF binding might contribute to contacts between putative enhancers and their target genes, as shown by many other previous studies^38^. In addition to enhancer regions, we observed a slight colocalization of the interaction-associated promoter-distal CTCF-binding sites with exons (7.5% versus 6.7% by chance, *P* = 1.8 × 10^−6^; Extended Data Fig. 3k), which is consistent with a role of CTCF in mediating promoter–exon interactions as previously reported^39^.

### Promoter-centered interactome shows tissue-to-tissue variability and developmental dynamics that correlate with gene expression

**Fig. 2:**
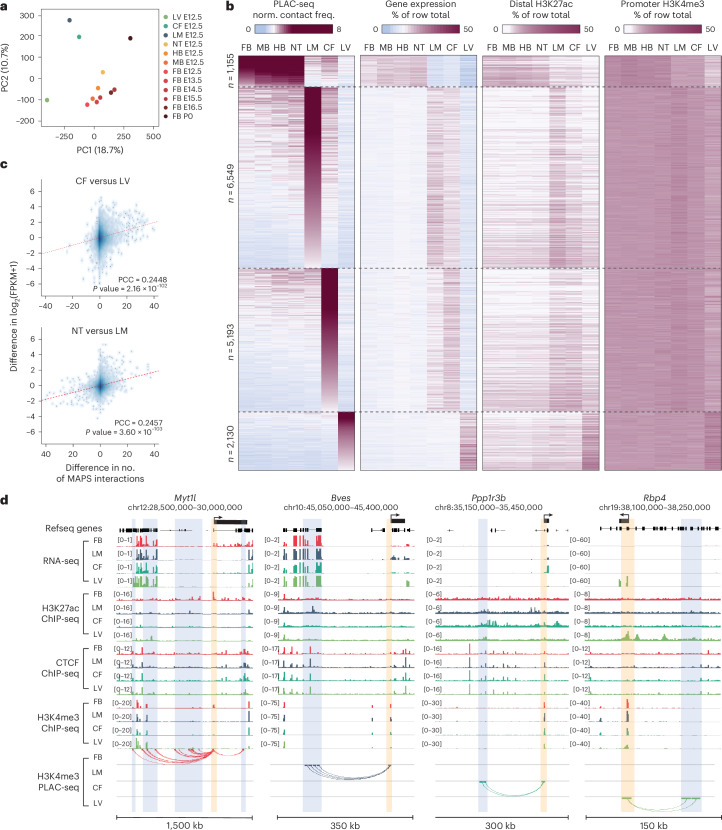
Tissue-to-tissue variability and developmental dynamics of promoter-centered interactomes. **a**, PCA for the normalized PLAC-seq contact frequency. **b**, Heat map displaying normalized contact frequencies (left), gene expression of interacting promoters (middle left), H3K27ac distal peak signal (middle right) and promoter H3K4me3 signal (right) in P2N tissue-specific interactions. % of row total: individual tissue percentage of total sum across all tissues. **c**, Scatter plot between the difference of interaction number and the difference in gene expression. The red dashed line represents the fitted linear line. **d**, Interactions anchored at TSS regions around four genes (highlighted by yellow boxes) in tissues at E12.5. Black boxes above the refseq gene track mark the gene boundary of each anchored genes and the arrows on the top represent their transcription direction. Source data

**Extended Data Fig. 4:**
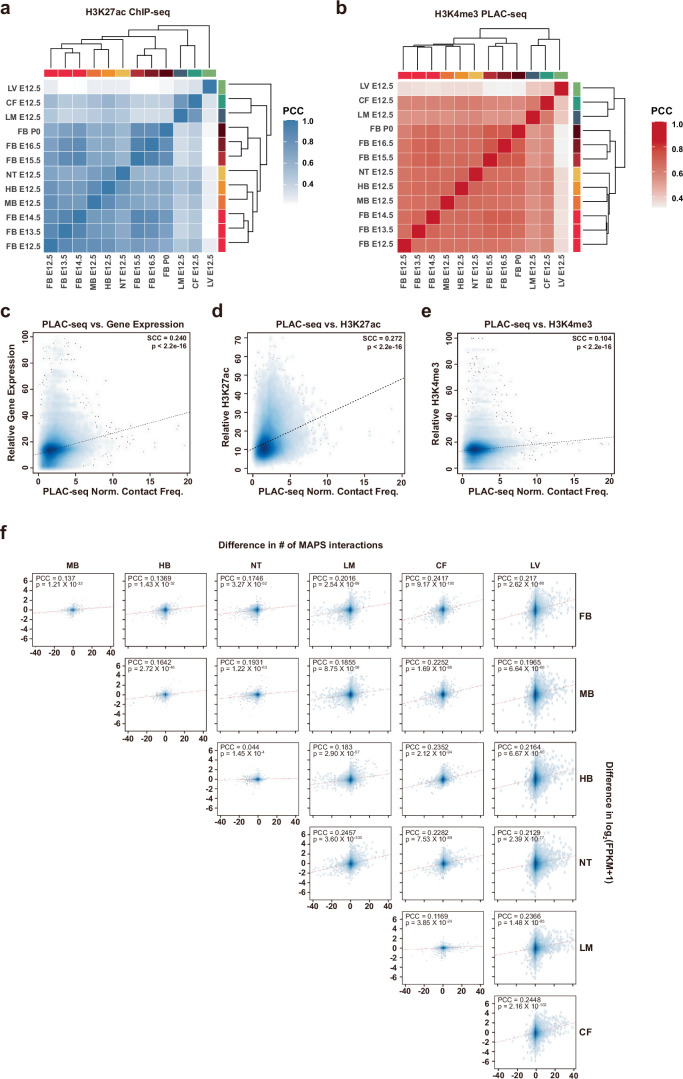
Tissue-specific chromatin interactions are associated with tissue-specific gene expression. **a**, **b**. Heat maps showing the Pearson correlation coefficients and hierarchical clustering of H3K27ac ChIP-seq (**a**) and normalized contact frequencies from H3K4me3 PLAC-seq (**b**), with color bars representing different tissues. **c**–**e**. Scatterplot between PLAC-seq normalized contact frequencies and relative gene expression (percent of total FPKM) (**c**), relative H3K27ac (percent of total log_2_CPM) (**d**) and relative H3K4me3 (percent of total log_2_CPM) (**e**) for interactions in Fig. 2b. **f**. Similar to Fig. 2c, scatterplot between the difference of the number of MAPS-identified significant chromatin interactions (x-axis) and the difference in gene expression (y-axis, merged by log_2_(FPKM + 1)), for all 21 pairs of 7 tissues at E12.5. The red dashed line represents the fitted linear line. All p-values are from two-sided Pearson or Spearman correlation test as indicated in figures.

To characterize the spatiotemporal dynamics of the promoter-centered interactomes during mouse fetal development, we performed principal component analysis (PCA) using normalized PLAC-seq contact frequencies and found that tissues with similar lineages or developmental stages clustered more closely (Fig. 2a), consistent with the hierarchical clustering results based on H3K4me3 PLAC-seq data and H3K27ac ChIP-seq data (Extended Data Fig. 4a,b).

We then evaluated the variability of promoter-centered interactomes across seven tissues at E12.5 and asked how the tissue-specific chromatin interactions are associated with tissue-specific epigenome and gene expression. For this purpose, we selected a total of 15,027 P2N interactions that were identified only in the neural tissues (including FB, MB, HB and NT), in LM, in CF or in LV (10.1% of all P2N interactions for tissues at E12.5), involving a total of 1,826 unique genes. We found that the normalized contact frequency of these tissue-restricted P2N interactions significantly correlated with gene expression and the H3K27ac signals on the promoter-distal elements (Fig. 2b and Extended Data Fig. 4c–e). Moreover, we compared the promoter-centered interactomes between each pair of tissues at E12.5 and found that the difference in the number of interactions for each promoter was positively correlated with the changes in gene expression of the corresponding tissues (Fig. 2c and Extended Data Fig. 4f). This trend was particularly pronounced between tissues of different developmental origins. For example, several neural-specific interactions were found at the promoter of *Myt1l*, a gene that has a key role in neuronal differentiation^40^. Regions interacting with the *Myt1l* promoter showed higher H3K27ac in the FB at E12.5 compared to the other three tissues (Fig. 2d and Supplementary Fig. 1). We also observed LM-specific, CF-specific and LV-specific interactions centered at the promoter of *Ppp1r3b* (a regulator of glycogen synthesis)^41^, *Bves* (a gene encoding blood vessel and epicardial substance, which may have a role in skeletal muscle development)^42^ and *Rbp4* (a retinol carrier that delivers retinol from LV to peripheral tissues)^43^, respectively, with high levels of H3K27ac on the promoter-interacting regions in the corresponding tissues (Fig. 2d and Supplementary Fig. 1).

**Extended Data Fig. 5:**
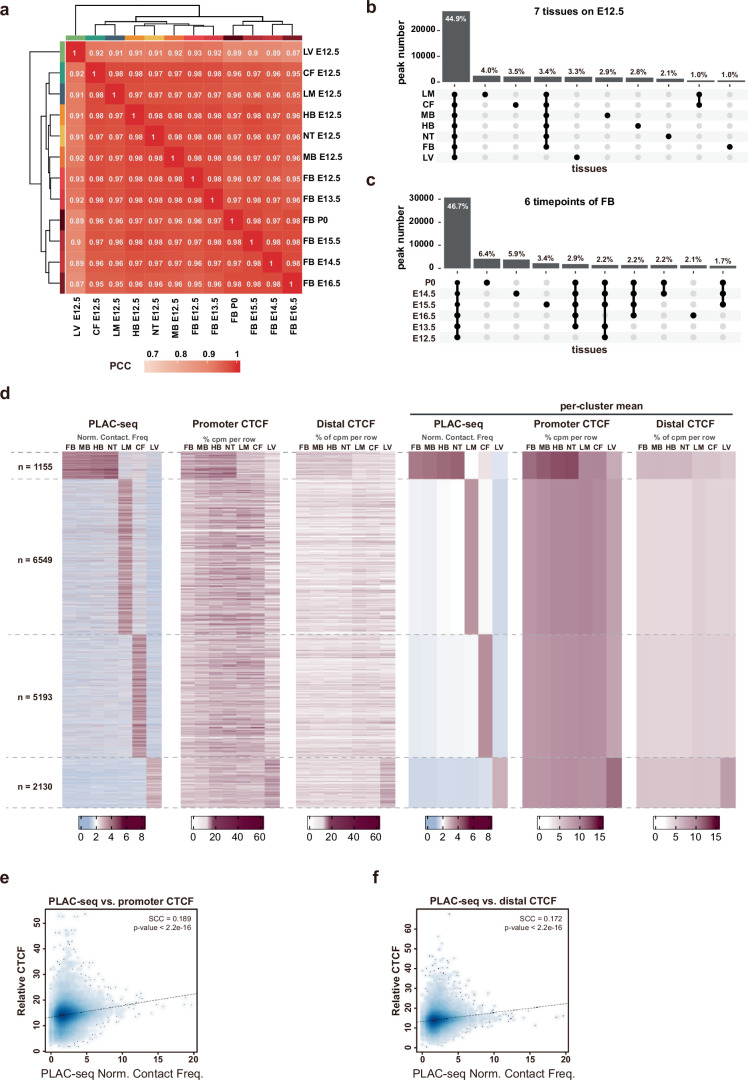
Correlation between dynamic CTCF and interactions across different tissues. **a**. Heat map showing the Pearson correlation coefficients and hierarchical clustering of CTCF ChIP-seq with color bars representing different tissues. **b**, **c**. Upset plots showing the numbers and the fractions of CTCF peaks shared by the different combinations of 7 tissues from embryonic day 12.5 (E12.5) (**b**) or 6 forebrain tissues from different timepoint (**c**). Only the top 10 combinations that contain the most CTCF peaks are showed in the plot. **d**. Heatmap displaying normalized contact frequencies, CTCF peak signal in the promoter and the non-promoter side of tissue-specific Promoter-to-Non-Promoter (P2N) interactions. CTCF are represented as individual tissue percentage of total sum across all 7 tissues. Tissue-specific interactions are clustered for being exclusive in all brain- and NT, LM, CF, or LV. **e**. Scatterplot between PLAC-seq normalized contact frequencies and relative CTCF (percent of total log_2_CPM) binding on the Promoter side of tissue-specific Promoter-to-Non-Promoter (P2N) interactions in **d**. **f**. Scatterplot between PLAC-seq normalized contact frequencies and relative CTCF (percent of total log_2_CPM) binding on the non-Promoter side of tissue-specific P2N interactions in **d**. All p-values are from two-sided Spearman correlation test as indicated in figures.

Because CTCF binding occurred on at least one end for the majority of the identified interactions (Extended Data Fig. 3c,d), we further checked whether variable CTCF binding is also associated with dynamic promoter-centered interactomes among different tissues. It is worth noting that CTCF-binding patterns were largely invariant between samples as previously reported^44^ (Extended Data Fig. 5a); nearly half of the CTCF-binding sites were conserved and variable CTCF occupancy only occurred at a small number of loci (Extended Data Fig. 5b,c). Nevertheless, we still observed a positive correlation between the normalized contact frequency and the promoter-proximal CTCF-binding signals (Spearman’s correlation coefficient (SCC) = 0.189, *P* < 2.2 × 10^−16^) and distal CTCF-binding signals (SCC = 0.172, *P* < 2.2 × 10^−16^) (Extended Data Fig. 5d–f).

**Extended Data Fig. 6:**
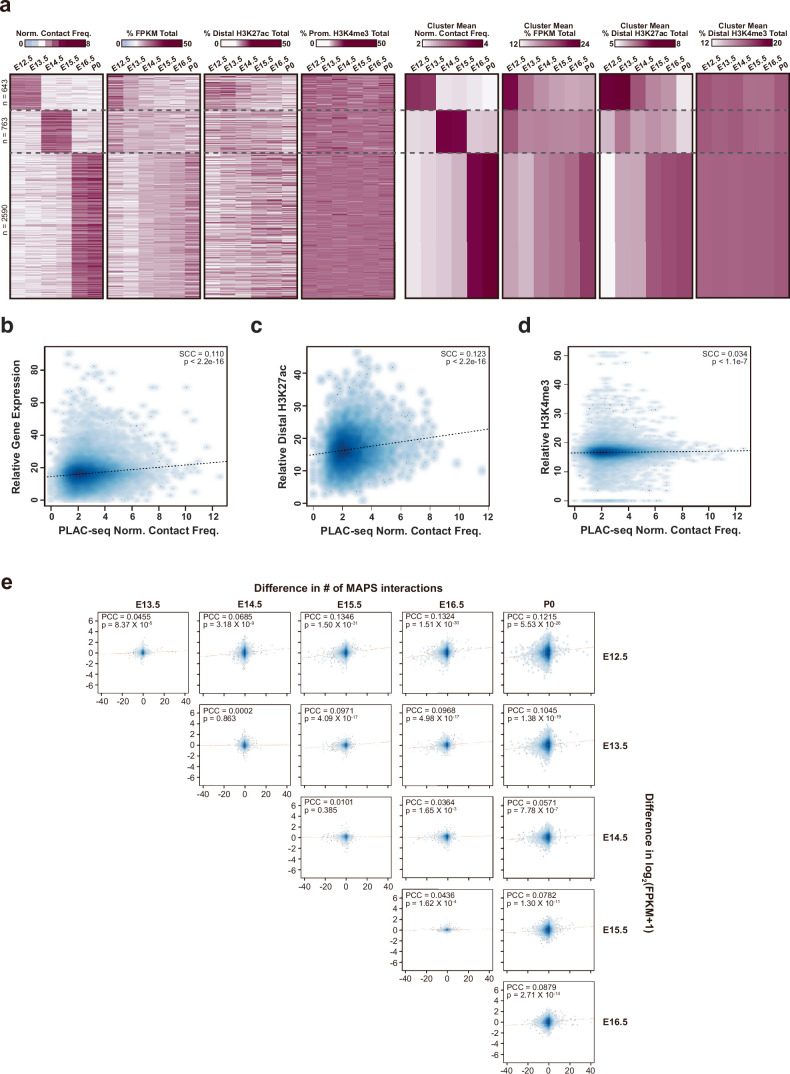
Dynamics of promoter-centered interactions across developmental stages in forebrain. **a**. Heat map displaying normalized contact frequencies, gene expression of interacting promoters, H3K27ac distal peak signal and promoter H3K4me3 signal in Promoter-to-Non-Promoter (P2N) stage-specific interactions (left) as well as their average value across each cluster in each tissue (right). Gene expression and H3K27ac are represented as individual tissue percentage of total sum across all tissues. Stage-specific interactions are clustered for being exclusive in E12.5/E13.5, E14.5/E15.5, or E16.5/P0-only interactions. **b**. Scatterplot between PLAC-seq normalized contact frequencies and relative gene expression (percent of total FPKM) for Promoter-to-Non-Promoter (P2N) interactions in **a**. **c**. Scatterplot between PLAC-seq normalized contact frequencies and relative H3K27ac (percent of total log2 CPM) for P2N interactions in **a**. **d**. Scatterplot between PLAC-seq normalized contact frequencies and relative H3K4me3 (percent of total log2 CPM) for P2N interactions in **a**. **e**. Similar to Fig. 2c, scatterplot between the difference of the number of MAPS-identified significant chromatin interactions (x-axis) and the different of gene expression (y-axis, merged by log2(FPKM + 1)), for all 15 pairs of forebrain tissue at 6 developmental stages. The red dashed line represents the fitted linear line, suggesting that the change of significant chromatin interactions is positively correlated with the change of gene expression. All P-values from two-sided Spearman correlation test as indicated in figure. Source data

We also examined the dynamics across developmental stages in the FB. We selected the E12.5–E13.5 FB-restricted, E14.5–E15.5 FB-restricted and E16.5–P0 FB-restricted P2N interactions and performed a similar analysis to that for tissue-restricted P2N interactions at E12.5 (Extended Data Fig. 6a–e). A positive correlation between interaction strength and gene expression was also observed, albeit weaker than that between different tissues at E12.5, suggesting that the developmental dynamics of the promoter interactome in general is less pronounced than the tissue-to-tissue variations. Taken together, our data demonstrate that the tissue-to-tissue variability and developmental dynamics of the promoter-centered interactome are correlated with the fetal gene expression programs.

### Chromatin contacts at promoters coupled with chromatin state maps predict target genes of distal cEnhancers

**Fig. 3:**
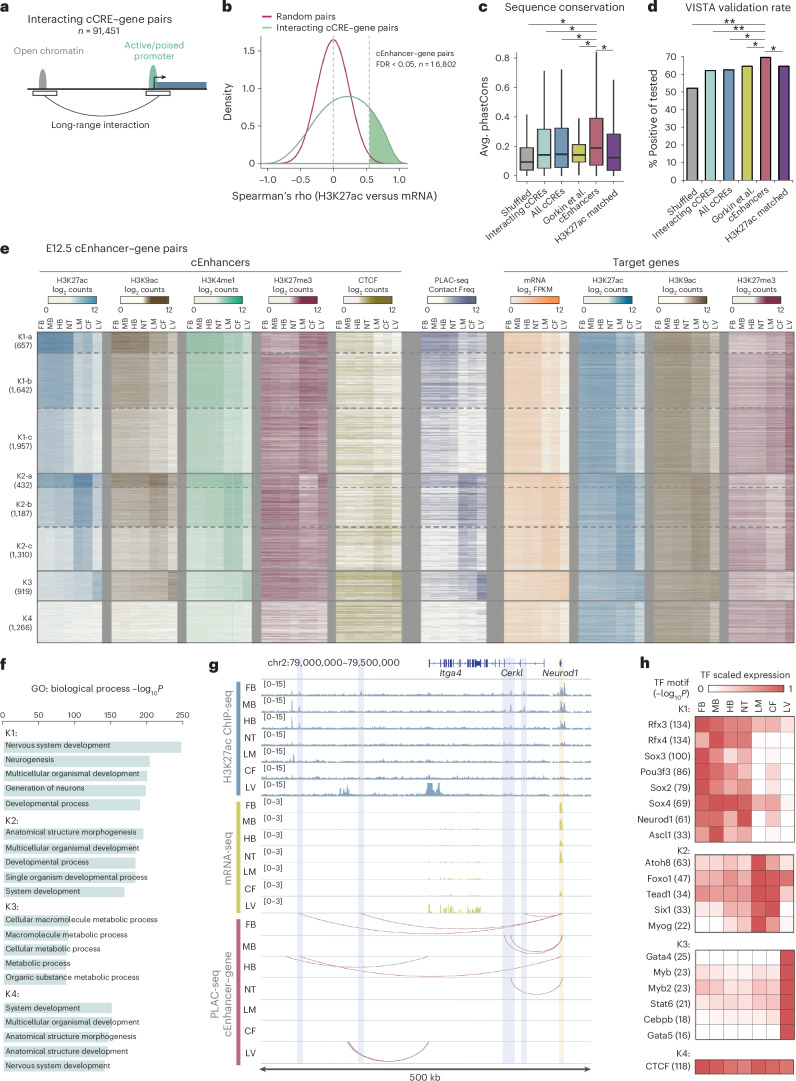
Profiling cEnhancer–gene pairs in different fetal tissues. **a**, Schematic for assigning cCREs to target genes. **b**, Density plot of SCCs between cCRE H3K27ac and interacting gene mRNA. **c**, Box plots of distributions for average phastCons scores for each nucleotide of all cCREs (d-TACs^8^, with a chromatin-accessible peak present in any of the 12 samples where PLAC-seq data were generated), size-matched random regions (shuffled), interacting cCREs (cCREs involved in 91,451 cCRE–gene pairs), predicted enhancers from Supplementary Table 8c of Gorkin et al.^8^, all cEnhancers or cCREs with H3K27ac levels matching cEnhancers (matched H3K27ac). Two-tailed Wilcoxon rank-sum test: *P < 2.2 × 10^−16^. Central bar, median; lower and upper box limits, 25th and 75th percentiles, respectively; whiskers, minimum and maximum value within the range of (first quartile − 1.5 × (third quartile − first quartile)) to (third quartile + 1.5 × (third quartile − first quartile)). **d**, Validation rate of indicated elements tested for in vivo enhancer activity from the VISTA elements database. Chi-square test: **P < 0.001 and *P < 0.05. **e**, Heat map for chromatin features and expression of E12.5 cEnhancer–gene pairs. Pairs were *k*-means clustered by H3K27ac signal surrounding 2 kb at the center of cEnhancer. **f**, Top enriched GO biological process terms for genes from clusters in **e**. **g**, Example gene, *Neurod1*, showing correlated E12.5 tissue-specific H3K27ac signal at cEnhancer, gene expression and cEnhancer–gene interactions. The cEnhancer–gene interactions in this region from each E12.5 tissue are marked by arcs. The *Neurod1* TSS region is highlighted by a yellow box and the cEnhancers are highlighted by blue boxes. **h**, Enriched known and de novo motifs at cEnhancers that have TF gene expression matching cEnhancer H3K27ac tissue patterns.

**Extended Data Fig. 7:**
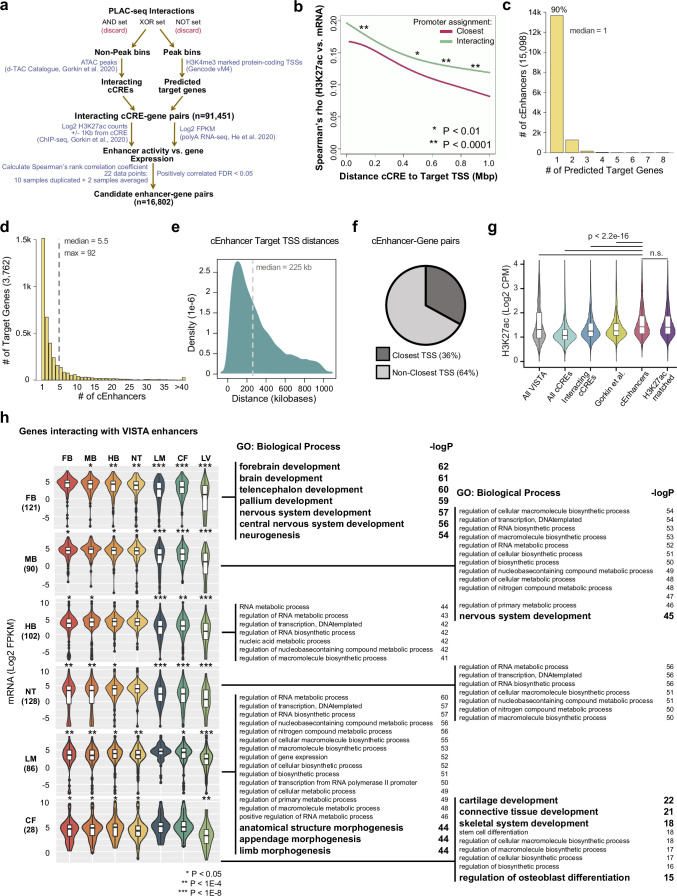
Chromatin interactions identify enhancer target genes. **a**. Schematic illustrating analysis pipeline for determining interacting cCRE-gene pairs and cEnhancer-gene pairs. **b**. Spline fit lines of Spearman’s correlation coefficients between cCRE H3K27ac and assigned gene mRNA as a function of cCRE to TSS distance for two different pairing strategies. Degrees of freedom = 2, Two tailed two-sample T-test for each 200 Kb interval without multiple comparison adjustment. **c**. Histogram for number of target genes assigned to cEnhancers. **d**. Histogram for number of cEnhancers assigned to target genes. **e**. Density plot of cEnhancer to target TSS distances. **f**. Pie chart representing percentage of pairs where the cEnhancer targets the gene with the closest TSS or not. **g**. Distribution of H3K27ac log2 counts per million (CPM) ± 1 kb from center of elements from each group in Fig. 3c, d. P-values from two-tailed student t-test without multiple comparison adjustment. **h**. Expression and top enriched GO: Biological Process terms for genes interacting with VISTA enhancers with an identified promoter interaction in the same tissue where enhancer activity was reported positive in the VISTA database. Due to low numbers of liver positive VISTA enhancers, only one gene (*Igf2*) was assigned to this group and was omitted from the figure. Paired T-test, two-sided, without multiple comparison adjustment. For all boxplot in this figure: Central bar, median; lower and upper box limits, 25th and 75th percentiles, respectively; whiskers, minimum and maximum value within the rage of (first quartile – 1.5× (third quartile – first quartile)) to (third quartile + 1.5 × (third quartile – first quartile)).

Long-range interactions between promoters and distal enhancers are believed to contribute to gene expression. However, a recent comprehensive study covering 24 diverse human cell types^23^ found that the variations in long-range interactions were only modestly correlated with the variations in gene expression, suggesting a complex relationship between the physical contacts of cCRE–gene pairs and gene expression. Recently, success has been achieved by combining the 3D chromatin information and chromatin state to predict the functional contributions of enhancers in gene expression^45,46^. To assign candidate enhancers (cEnhancers) to their target genes in mouse fetal tissues, we leveraged the P2N interactions identified from H3K4me3 PLAC-seq data. Using the chromatin interactions, open chromatin regions and H3K4me3 ChIP-seq peaks from the same sample, we first identified 91,451 open chromatin sites to be in long-range contact with active or poised promoters of protein-coding genes, designated as interacting cCRE–gene pairs (Fig. 3a and Extended Data Fig. 7a).

The H3K27ac signal at distal enhancers has been shown to correlate with the expression level of their target genes^45,47^. Assuming that a subset of these gene-interacting cCREs are enhancers, we performed the correlation analysis to determine which cCREs likely function as enhancers to their interacting genes. Specifically, we calculated the SCCs between the H3K27ac signal at the distal cCRE with gene expression levels of the interacting gene across 22 replicates of tissue–time point combinations (Supplementary Table 3). The distribution for correlations between H3K27ac signal at cCRE and mRNA-seq from the interacting genes was not only higher than that between randomly shuffled pairs (Fig. 3b) but also higher than that between cCREs and their closest promoters (Extended Data Fig. 7b), supporting an active role for 3D chromatin interactions in gene regulation. We classified 16,802 cEnhancer–target gene pairs as those that had a positive correlation (false discovery rate (FDR) < 0.05) (Fig. 3b, Extended Data Fig. 7a and Supplementary Table 4). Of the total 15,098 cEnhancers identified in these pairs, 90% had one predicted target gene (Extended Data Fig. 7c), while most predicted target genes had multiple assigned enhancers (Extended Data Fig. 7d). Most cEnhancers were predicted to target promoters more than 200 kb away (Extended Data Fig. 7e), with 64% of them targeting a gene that was not the nearest promoter (Extended Data Fig. 7f). Further supporting a role for cEnhancers predicted to regulate target genes in the mouse embryonic tissues, we found that they had higher sequence conservation than either a random set of size-matched genomic regions or the full list of cCREs (‘Shuffled’ and ‘All cCREs’ in Fig. 3c). Importantly, they also had higher sequence conservation than the subset of cCREs predicted to regulate target genes based on only correlated ChIP-seq and RNA-seq signals without consideration of 3D interaction data (‘Gorkin et al.’ in Fig. 3c)^8^. To further assess the validity of these enhancers, we compared them to elements in the VISTA database^48^. VISTA elements are experimentally tested for enhancer activity by an in vivo reporter assay in E11.5 or E12.5 mouse embryos. Referencing the VISTA database, we found that the above enhancers had a significantly higher rate for positive enhancer activity than all cCREs, interacting cCREs and enhancers predicted without considering 3D interactions (Fig. 3d). Because the H3K27ac levels on the cEnhancers were significantly higher than all abovementioned control sets (Extended Data Fig. 7g), we further included a set of cCREs with H3K27ac levels matching cEnhancers as additional control (‘H3K27ac-matched’). Again, both the sequence conservation and the VISTA validation rate of cEnhancers were significantly higher than those of the H3K27ac-matched cCREs (Fig. 3c,d). Taken together, our list of cEnhancers is supported by multiple lines of evidence for their role in spatiotemporal expression in the mouse embryos. While both chromatin state–gene expression correlation and 3D chromatin interactions have been widely applied to predict cCRE–gene pairs^31,49,50^, efforts that integrate both types of information are rare. Our analyses highlight the power of integrating cCRE–gene activity correlation data with 3D interaction information for enhancer prediction.

Cell-type-specific and tissue-specific gene expression is often dependent on enhancers for activation^51^; therefore, we hypothesized that genes interacting with predicted cEnhancers would be highly expressed in tissues where they are active. Indeed, genes interacting with VISTA enhancers through the above enhancer–gene links were expressed relatively highly in the same tissue where enhancer activity was reported; the enriched Gene Ontology (GO) terms for genes interacting with VISTA enhancers were related to the tissue where the VISTA enhancer was active (Extended Data Fig. 7h). These results support a functional relationship between interacting enhancers and promoters and demonstrate the importance of these interactions for tissue-specific activation of gene expression during mouse fetal development.

### Tissue-specific gene-regulatory programs during mouse fetal development

**Extended Data Fig. 8:**
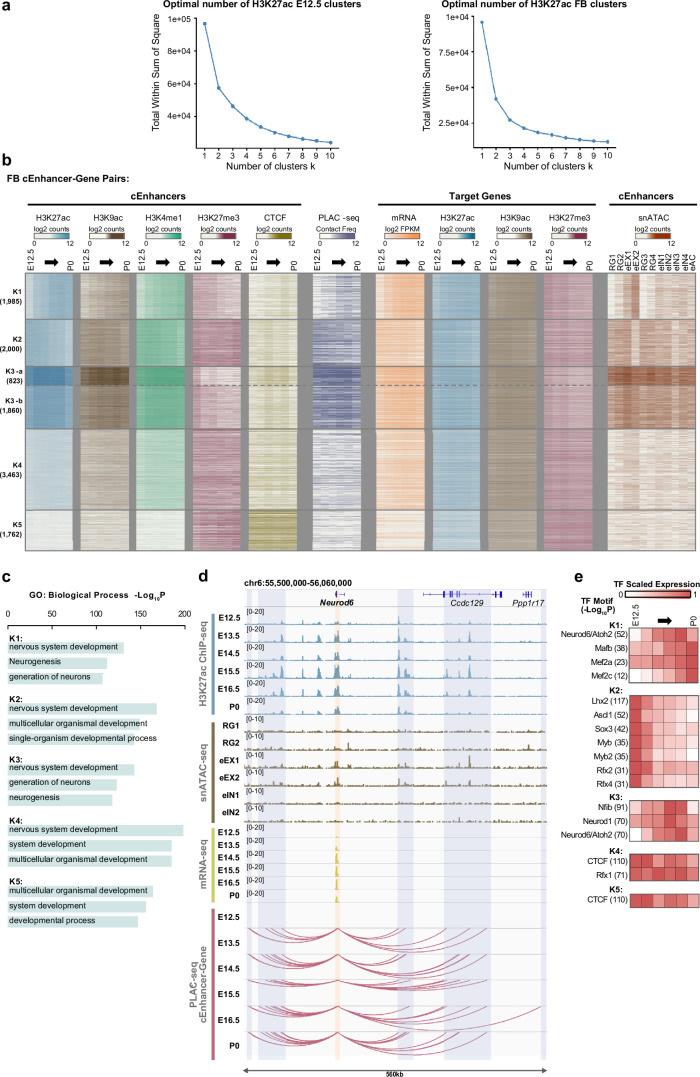
Profiling candidate forebrain enhancer-gene pairs across developmental stages. **a**. Elbow plots displaying sum of squared distances for indicated number of Kmeans clusters. Kmeans clustering was performed using normalized H3K27ac counts surrounding 2 Kb of cEnhancers of cEnhancer-gene pairs present in E12.5 tissues (left) and FB developmental stages (right). **b**. Heat map for chromatin features and expression of forebrain cEnhancer-gene pairs. Pairs were k-means clustered by H3K27ac signal surrounding 2 Kb at center of cEnhancer. **c**. Top enriched GO: Biological Process terms for genes of clusters from **b**. **d**. Example gene, *Neurod6*, showing correlated stage-specific cEnhancer H3K27ac, gene expression, and cEnhancer-gene interactions. The cEnhancer-gene interactions in this region from FB at different stages were marked by arcs. *Neurod6* TSS region was highlighted by yellow box and the cEnhancers are highlighted by blue boxes. **e**. Enriched known and *de novo* motifs at cEnhancers that have TF gene expression matching cEnhancer H3K27ac patterns across stages.

To investigate the tissue-specific gene expression programs in mouse embryos, we focused on the predicted cEnhancer–gene pairs. We performed *k*-means clustering of H3K27ac signals at the above cEnhancers for pairs that had a significant PLAC-seq interaction in one or more E12.5 tissues. These pairs were clustered into four main groups (K1–K4) with tissue-specific H3K27ac signals (Fig. 3e and Extended Data Fig. 8a). cEnhancers in clusters K1, K2 and K3 were marked with high H3K27ac signals in neural tissues, LM and CF tissues, and LV tissues, respectively, while cEnhancers in cluster K4 had low H3K27ac signals across all seven tissues but relatively high CTCF signals. Clusters K1 and K2 were subclustered into K1-a, K1-b, K1-c, K2-a, K2-b and K2-c on the basis of the overall H3K27ac signal strength (Fig. 3e). When examining the additional histone modifications at cEnhancers, we observed a high degree of correlation among H3K27ac, H3K9ac and H3K4me1, which had a negative correlation with repressive mark H3K27me3 (Supplementary Fig. 2a). Similar to H3K27ac, these additional histone marks displayed highly tissue-specific patterns and further support cEnhancers as putative functional regulatory elements.

To determine the degree of tissue specificity for long-range chromatin interactions at E12.5 cEnhancer–gene pairs, we plotted normalized contact frequencies (Fig. 3e). Tissue-specific contact strengths strongly correlated with cEnhancer H3K27ac signal (Fig. 3e and Supplementary Fig. 2a), demonstrating that enhancer activity correlates with the strength of long-range promoter-anchored chromatin interactions. Conversely, there was a strong negative correlation between cEnhancer H3K27me3 signal and contact strength (Fig. 3e and Supplementary Fig. 2b), suggesting that Polycomb-mediated repression may function to prevent aberrant enhancer–promoter contacts for proper tissue specification. Additionally, target genes regulated by E12.5 cEnhancers demonstrated tissue-specific expression and active histone marks at their promoter regions (Fig. 3e). Expression levels negatively correlated with H3K27me3 signal at promoter regions (Fig. 3e and Supplementary Fig. 2b), suggestive of pervasive Polycomb-mediated repression of tissue-specific gene expression by promoter silencing in addition to distal enhancer silencing. Taken together, this integrative analysis showcases the high degree of tissue-specific activity of enhancer–promoter pairs during mouse fetal development, indicating the potential regulatory function of such enhancer–promoter pairs as previously reported in human tissues^18,19^.

GO analysis of cEnhancer target genes from E12.5 clusters revealed high enrichment for tissue-specific developmental genes (Fig. 3f). For example, genes targeted by cEnhancers active in neural tissues (K1) had the highest enrichment for terms related to nervous system development (*P* = 1 × 10^−255^). Genes targeted by cluster K2 cEnhancers active in LM and CF were related to anatomical structure morphogenesis (*P* = 1 × 10^−196^), while genes targeted by LV-active cEnhancers functioned in the cellular macromolecule metabolic process (*P* = 1 × 10^−96^). These data demonstrate the high degree of tissue specificity of long-range enhancer–promoter interactions for tissue-determining gene expression during mouse fetal development. For example, *Neurod1*, which encodes a brain-specific TF involved in neuron differentiation, was selectively expressed in the nervous system and was assigned to distal cEnhancers only in FB, MB, HB and NT (Fig. 3g).

To identify potential developmental regulators involved in establishing cEnhancers, we performed TF motif enrichment analysis for these cEnhancers. We found highly enriched TF motifs for each cluster and filtered the top enriched TFs for those with tissue expression patterns that matched with cEnhancer activity (Fig. 3h). This analysis produced a high-confidence list of TFs that likely activate tissue-specific developmental enhancers and many known developmental regulators were identified in the expected clusters. For example, K1 was enriched for RFX family, SOX family, POU3f3, NEUROD1 and ASCL1 TF motifs, all of which have been previously reported to have important roles in nervous system development^52–56^. FOXO1 and ATOH8, important for chondrogenic commitment of skeletal progenitor cells^57,58^, and MYOG and SIX1, important for skeletal muscle fiber differentiation^59^, were enriched at K2 cEnhancers active in LM and CF. LV-specific cEnhancers had enrichment for hepatocyte differentiation factors GATA4 and CEBPB (ref. ^60^). Lastly, we found that the CTCF motif had the highest enrichment for K4 cEnhancers, supporting the high CTCF binding detected by ChIP-seq at these cEnhancers (Fig. 3e). Our results implicate that several TFs to bind distal enhancers that physically interact with target promoters and activate tissue-specific gene expression.

### Dynamic enhancer–promoter interactions in developing FB

We also performed *k*-means clustering of the H3K27ac signal at the cEnhancers from cEnhancer–gene pairs with a significant PLAC-seq interaction present in the FB between E12.5 and P0 and produced five clusters (Extended Data Fig. 8a), with K1, K2 and K3 showing highly dynamic changes in active and repressive histone marks through developmental stages (Extended Data Fig. 8b). Interestingly, the chromatin interaction strengths were also dynamic across developmental stages in a pattern similar to H3K27ac. Consistent with E12.5 clusters (Fig. 3e), active histone marks at promoters correlated with those at interacting cEnhancers though with less overall dynamics (Supplementary Fig. 2c). We then integrated previously generated single-nucleus ATAC-seq data from the same fetal mouse FB samples^61^ to identify specific FB cell types contributing to the cEnhancer activity from these clusters (Extended Data Fig. 8b). Cluster K1 cEnhancers gained signal for active histone marks as development progressed, which appeared to be contributed mostly by eEX2 excitatory neurons. Excitatory neurons show low abundance at stage E12.5 but become more abundant as development progresses. In particular, eEX2 cells are the most abundant cell type at P0 (ref. ^61^), suggesting that eEX2 abundance contributes to these tissue-wide H3K27ac dynamics at K1 cEnhancers. Conversely, H3K27ac at K2 cEnhancers gradually decreased from E12.5 to P0, which reflects these sites having closed, inactive chromatin in eEX2 cells (Extended Data Fig. 8b). The integration of cell-type-specific open chromatin data demonstrates that FB enhancer dynamics during fetal development is correlated with changes in cellular composition.

We performed GO term analysis on genes from each FB cEnhancer–gene pair cluster. For clusters K1–K4, the most enriched term was nervous system development, while cluster K5, which had low H3K27ac signal and high CTCF binding at cEnhancers at all developmental stages, displayed GO enriched terms related to development without specific tissue designation (Extended Data Fig. 8c). An example of a gene with temporally dynamic enhancer–promoter interactions is *Neurod6*, which encodes a TF important for neuronal differentiation^62^. Expression of *Neurod6* is low at E12.5, where there are no assigned interacting cEnhancers, but becomes activated at E13.5, coinciding with the onset of several cEnhancer interactions at H3K27 acetylated distal sites (Extended Data Fig. 8d). These distal-interacting cEnhancers are marked with open chromatin in excitatory neurons, cells known for high *Neurod6* expression^63^. TF motifs enriched at cEnhancers with TF expression patterns that match cEnhancer activity implicated many known brain developmental regulators as activators for these enhancers (Extended Data Fig. 8e). Our results demonstrate that the extensive temporal dynamics of enhancer–promoter interactions regulate the precision of stage-specific gene expression during mouse fetal brain development.

### Enhancer–promoter interactions facilitate interpretation of noncoding risk variants in the human genome

**Extended Data Fig. 9:**
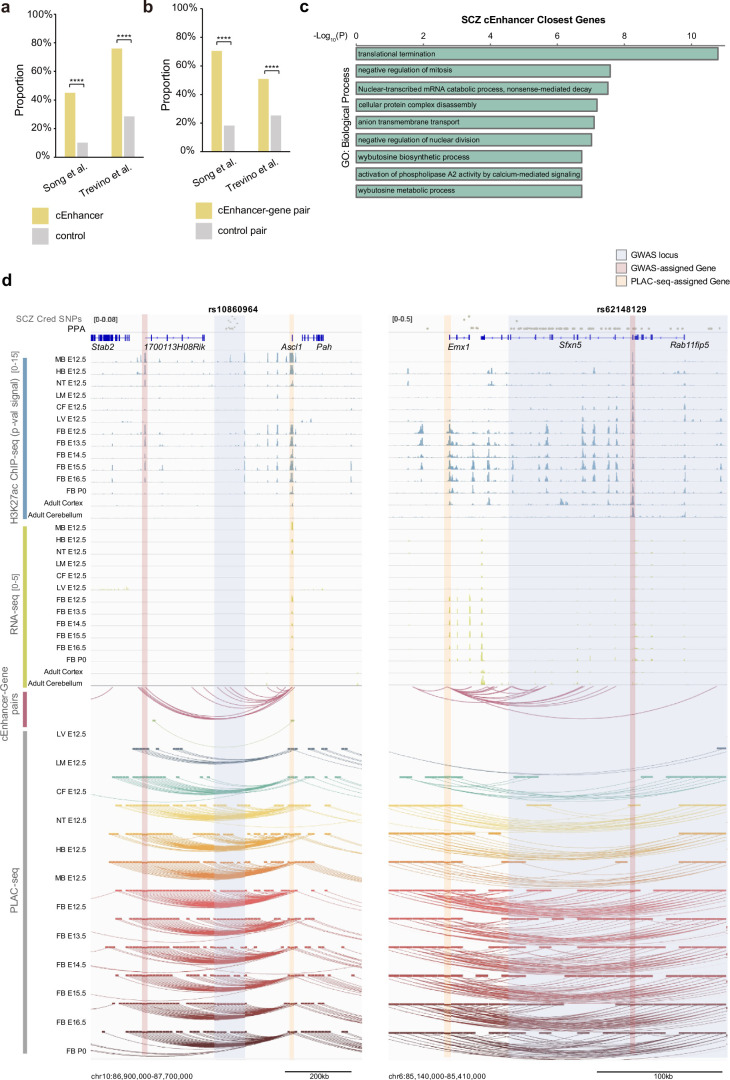
SCZ-associated non-coding variants are enriched at fetal-specific enhancers. **a**. Proportions of cEnhancer orthologs re-captured by CREs from two human fetal cortex datasets (Song et al.^21^ and Trevino et al.^65^). Regions of equal size to the cEnhancer ortholog, with equidistant distance from but on the other side of the target gene were used as control. Chi-square test, **** p < 2.2e-16. **b**. Proportions of cEnhancer-gene orthologs supported by the two human datasets^21,65^. Pseudo-pairs linking the target genes with the control regions were used as controls. Chi-square test, **** p < 2.2e-16. **c**. Gene ontology analysis showing top enriched Biological Process terms of genes closest to cEnhancers harboring SCZ credible set SNPs by TSS proximity at 1D genomic distance. P-values from two-sided Fisher’s exact test. **d**. SCZ example genes, *Ascl1* and *Emx1*, showing correlated tissue-specific cEnhancer H3K27ac, gene expression, and chromatin interactions at loci harboring SCZ SNPs.

Genome-wide association studies (GWASs) have identified hundreds of thousands of common genetic variants linked to various traits and diseases. However, most GWAS variants reside in noncoding regions and it is still very challenging to interpret their functions^64^. To address this challenge, we used our list of cEnhancer–gene pairs in the mouse genome to predict gene targets for human enhancers harboring noncoding GWAS variants. We identified 12,929 human orthologous regions corresponding to the 15,098 mouse cEnhancers (Supplementary Table 5), of which 68% overlapped with human cCREs in the ENCODE registry (https://screen.encodeproject.org). To verify the reliability of such cross-species comparison, we further compared our cEnhancers and cEnhancer–gene ortholog pairs with two sets of previously reported developmental cCREs and cCRE–gene pairs identified from human fetal brains and found high overlap^21,65^ (Extended Data Fig. 9a,b), suggesting that the mouse fetal enhancers may provide valuable information to help interpret the GWAS variants.

**Fig. 4:**
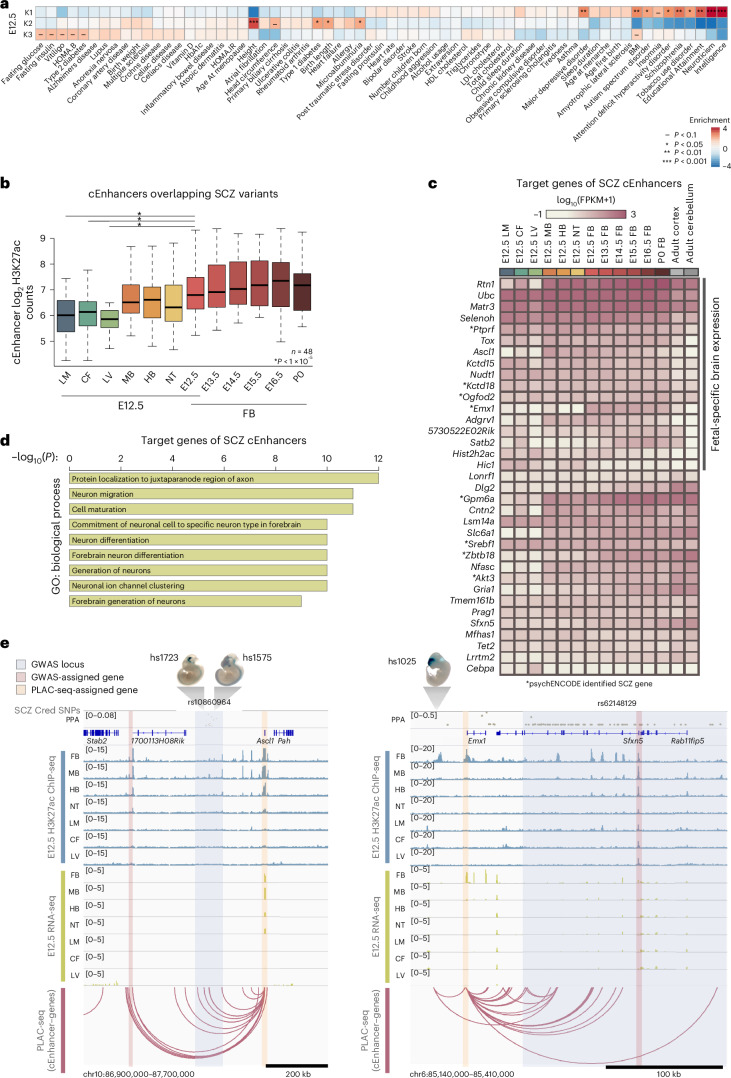
Chromatin interactions facilitate interpretation of noncoding disease risk variations in the human genome. **a**, LDSC regression analysis to identify GWAS enrichments at cEnhancers from E12.5 clusters shown in Fig. 3e. **b**, Box plots showing distribution of H3K27ac counts at cEnhancers overlapping SCZ credible set SNPs. Two-tailed paired *t*-test between E12.5 FB and non-neural tissues. Central bar, median; lower and upper box limits, 25th and 75th percentiles, respectively; whiskers, minimum and maximum value within the range of (first quartile − 1.5 × (third quartile − first quartile)) to (third quartile + 1.5 × (third quartile − first quartile)). **c**, Heat map of gene expression for genes identified as interacting with cEnhancers overlapping SCZ credible set SNPs (*n* = 35); psychENCODE SCZ genes were marked by asterisk^51^. Adult mouse cortex and cerebellum RNA-seq data obtained from ENCODE. **d**, GO analysis showing top enriched biological process terms of genes interacting with cEnhancers harboring SCZ credible set SNPs. **e**, SCZ example genes, *Ascl1* and *Emx1*, interacting with loci harboring SCZ candidate SNPs. Tracks display H3K27ac, gene expression and all identified nearby cEnhancer–gene interactions. Loci are labeled with SNPs rs10860964 and rs62148129. Select images of mouse embryos displaying positive VISTA enhancer activity in the displayed genomic regions were obtained from VISTA database. All cEnhancer–gene pairs with a PLAC-seq interaction in any tissue or stage tested present in these genomic regions are marked by arcs.

Using these human orthologous regions, we performed linkage disequilibrium score (LDSC) regression on a panel of 65 GWAS phenotypes. Clusters of E12.5 active cEnhancers (Fig. 3e) were enriched for GWAS variants associated with specific phenotypes (Fig. 4a). In most cases, enrichments displayed tissue specificity that matched with tissues expected to contribute to the associated phenotype, indicating the potential of our annotated mouse enhancer–gene pairs for understanding mechanisms related to disease caused by noncoding genetic variants (Fig. 4a). For example, neural-active K1 cEnhancers were found enriched for noncoding variants associated with neurological disorders, K2 enhancers active in LM and CF were enriched for noncoding variants associated with physical size and K3 enhancers active in LV had the highest enrichment for noncoding variants associated with metabolic diseases.

Schizophrenia (SCZ) is a neurological condition in adulthood that is associated with many genetic variants; however, the disease etiology remains poorly understood^66^. To predict putative target genes of SCZ-associated noncoding variants, we focused on a 99% credible set of 20,373 SCZ single-nucleotide polymorphisms (SNPs)^67^ (obtained from https://pgc.unc.edu/) concentrated at 266 genomic loci. We identified 48 human orthologous cEnhancers containing SCZ-associated SNPs that target 35 genes (Fig. 4b,c and Supplementary Table 6). The mouse enhancers had high H3K27ac signal in neural tissues (Fig. 4b) and their predicted target genes displayed high levels of gene expression (Fig. 4c). The predicted target genes were highly enriched for GO terms related to neuronal differentiation (Fig. 4d) and included TFs, such as ASCL1, EMX1, TOX, and ZBTB18, known to regulate neuronal developmental (Fig. 4c). However, genes closest to these 48 mouse cEnhancers lacked brain-specific GO term enrichment (Extended Data Fig. 9c). Interestingly, 17 of the predicted target genes were expressed in fetal mouse brain but became repressed in adult brain (Fig. 4c), suggesting that aberrant gene expression during fetal brain development may contribute to SCZ, a disease with onset most common in early adulthood. Furthermore, many of our predicted target genes were not identified in studies using nonfetal human data^68^, demonstrating that fetal mouse enhancer–promoter interactions add additional insights into understanding molecular mechanisms contributing to SCZ.

**Extended Data Fig. 10:**
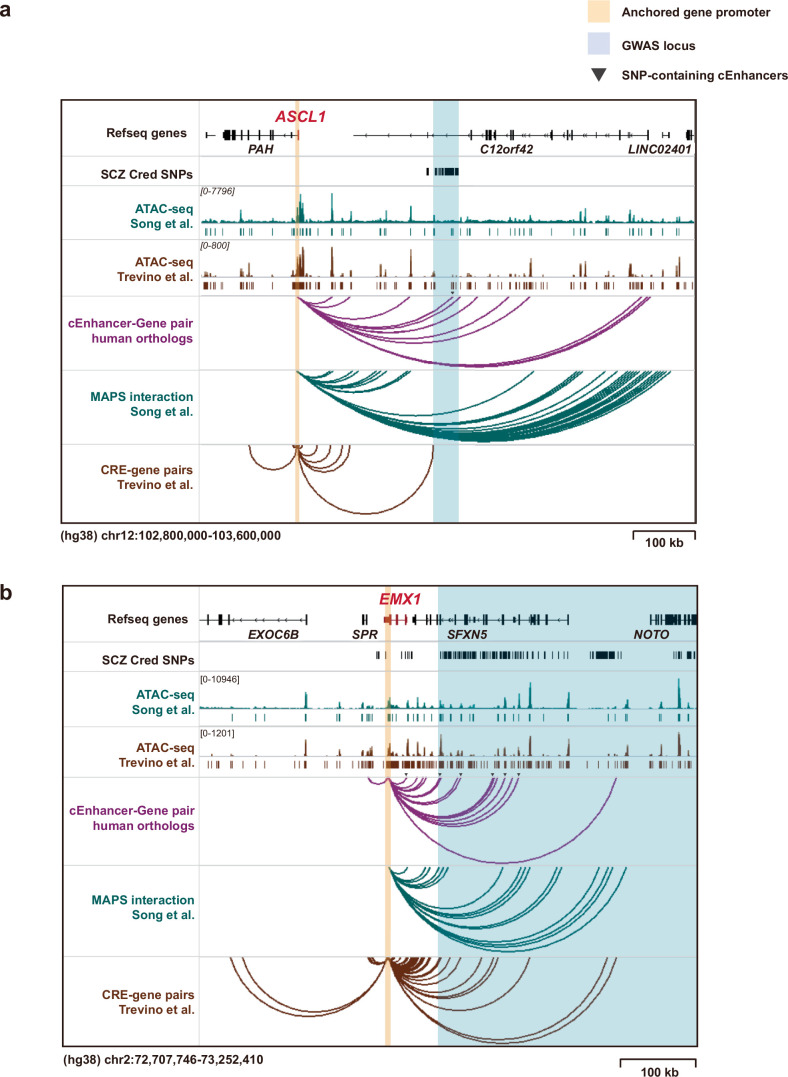
Examples of interactions between SCZ example genes and loci harboring SCZ-candidate SNPs in human datasets. IGV visualization of the human orthologs of cEnhancer-gene pairs of two SCZ example genes, *Ascl1* (**a**) and *Emx1* (**b**), under hg38 coordinate system. ATAC-seq signals from all 4 types of cells in Song et al.^21^ and ATAC-seq signals from 22 cell clusters in Trevino et al.^65^ are overlayed respectively and the corresponding ATAC-seq peaks are labelled on the bottom of each signal track.

For example, *Ascl1*, a regulator of neuroblast differentiation, forms long-range chromatin interactions in fetal mouse FB with cEnhancers, the human orthologous loci of which harbor SCZ SNPs. By proximity in genomic distances, the SCZ risk variants are assigned to the nearest and uncharacterized gene *C12orf42*; however, chromatin interaction data from the mouse FB revealed that the enhancers harboring these risk variants likely regulate *Ascl1* (Fig. 4e). Two VISTA elements confirm that this locus exhibits brain-specific and NT-specific enhancer activity during fetal development (Fig. 4e). Because of its fetal-specific enhancers and gene expression in brain (Extended Data Fig. 9d), *Ascl1* was not identified previously as a candidate SCZ causal gene^68^. The two recent studies using human fetal brains did not identify any target genes for this group of SCZ risk variants either, probably because the fetal samples were only available for limited time points^21,65^ (Extended Data Fig. 10a). Studies have consistently found abnormalities in thalamocortical axonal connectivity in SCZ patients^69^ and the thalamocortical network is critical for relaying sensory information from the periphery to the neocortex. Supporting the assignment of *Ascl1* as a target gene of the SCZ risk variants, *Ascl1*-null mice exhibited defects in thalamocortical axonal connectivity^70^. Such abnormalities in thalamocortical axonal connectivity are consistently found in SCZ patients and have been postulated to contribute to hallucination events^71^. Additionally, *Emx1*, a regulator of neuronal commitment, had high expression in fetal FB but was repressed in adult brain (Fig. 4c and Extended Data Fig. 9d). We identified *Emx1* promoter interacted with several cEnhancers marked with FB-specific H3K27ac including VISTA element hs1025, which displayed fetal enhancer activity exclusively in the FB (Fig. 4e). The human orthologous sequences of the *Emx1*-interacting cEnhancers harbor SCZ risk SNPs; however, by proximity, these risk variants are assigned to a different gene, *Sfxn5* (Fig. 4e), which encodes a widely expressed mitochondrial tricarboxylate carrier^72^. The pairing between *Emx1* gene and the region harboring SCZ risk SNPs is also supported by two recent studies with human fetal samples^21,65^ (Extended Data Fig. 10b). These examples showcase the value of our catalog of developmental enhancer–gene pair predictions for a molecular understanding of how genetic variation contributes to complex human disease.

## Discussion

In this study, we comprehensively profiled the promoter-centered chromatin interactomes in 12 mouse fetal tissues using H3K4me3 PLAC-seq and identified a total of 248,620 long-range chromatin interactions. In accordance with previous reports, the long-range interactions exhibited both tissue-to-tissue variability and developmental dynamics, while the promoter-interacting regions were enriched for tissue-specific open chromatins and enhancer marks. Integrating this dataset with CTCF ChIP-seq data also revealed a prevalent involvement of CTCF in facilitating promoter–enhancer contacts in various fetal tissues.

Annotating target genes of cEnhancers is a major challenge confronting geneticists today. First, accurately defining functional enhancers from epigenomic datasets alone is nontrivial; our previous study found that only ~30–60% of putative enhancers with known enhancer marks (such as H3K27ac signal) drive reporter genes in corresponding tissues in vivo in transgenic assays^8^. Second, deletion of an individual enhancer often does not notably alter the expression level of its target gene assigned by physical contacts, probably because the deleted enhancer is redundant or the detected enhancer–promoter contact is too weak or simply a false positive signal. To improve the accuracy in predicting functional enhancer–promoter pairs, we took advantage of the rich datasets from 12 mouse fetal tissues and integrated 3D chromatin interaction datasets with epigenomic and transcriptomic datasets for such prediction. We assumed that the functional enhancer–promoter pairs not only show significantly enriched physical contacts from our PLAC-seq data but also have positive correlation on their epigenomic signals and gene expression across different tissue and cell types. As a result, we found a total of 16,802 cEnhancer–gene pairs and further leveraged these pairs to assign noncoding genetic variants associated with various human diseases and traits. Specifically, the analysis on SCZ-associated noncoding variants revealed potential SCZ-associated genes that were exclusively expressed in fetal mouse brain but not identified in previous studies using nonfetal human data. Such analysis showcases the value of using mouse fetal omic data to reveal novel insights into human disease etiology.

Despite the comprehensive datasets included in this study, there are still a few important limitations. Firstly, the prediction of cEnhancer–gene pairs solely relied on the omics data; however, functional validations, such as the deletion of cEnhancers in relevant cell types or tissues followed by gene expression profiling, are necessary to experimentally validate their contribution to the target gene expression. Secondly, all the tissues used in this study were heterogeneous. Epigenome and 3D genome information at single-cell resolution is critical to further delineate the specific cell types contributing to the changes in cEnhancer activity and long-range interactions. For example, the activity changes on different groups of cEnhancers in the FB is likely to be caused by the cell composition changes during development according to the single-nucleus ATAC-seq data^61^ (Extended Data Fig. 8b). Lastly, not all the fetal tissues studied in ENCODE Phase III were included in this study because of an exhaustion of tissue samples. Nevertheless, we believe that the findings presented here demonstrate the potential of integrating multiomics datasets to discover new mechanistic insights into gene regulation. Such insights gained from early mouse fetal tissues may also be extrapolated to infer gene-regulatory networks influencing human development and disease.

## Methods

This study was performed in compliance with all relevant ethical regulations. All animal work was reviewed and approved by the Lawrence Berkeley National Laboratory Animal Welfare and Research Committee.

### Tissue collection

Tissue collection was performed from female C57BL/6N *Mus musculus*. All mouse fetal tissues used in this study were collected for the third phase of ENCODE^8^ and the step-by-step protocol can be found on the ENCODE project website (https://www.encodeproject.org/documents/631aa21c-8e48-467e-8cac-d40c875b3913/@@download/attachment/StandardTissueExcisionProtocol_02132017.pdf).

### Tissue fixation

First, 30–50 mg of tissues were crosslinked with 1% formaldehyde (v/v) in 1× PBS buffer with 0.1 M NaCl, 1 mM EDTA, 0.5 mM EGTA and 50 mM HEPES (pH 8.0) at room temperature for 15 min with slow rotation. The fixation was quenched by 0.125 M glycine with slow rotation at room temperature for 5 min. Fixed tissues were washed with ice-cold PBS, snap-frozen in liquid nitrogen and stored at −80 °C.

### H3K4me3 PLAC-seq data generation

First, 30–50 mg of frozen tissues were thawed on ice and then resuspended in 1 ml of lysis buffer (10 mM Tris-HCl pH 8.0, 5 mM CaCl_2_, 3 mM magnesium acetate, 2 mM EDTA, 0.5 mM EGTA, 1 mM DTT and 0.1 mM PMSF with proteinase inhibitor). Tissue dissociation was performed using the gentleMACS dissociator with program ‘Protein-M-tube-1.0’. After dissociation, the sample was filtered with a 40-µm cell strainer to remove large particles and additional lysis buffer with 0.4% Triton X-100 was added at an equal volume to make the final concentration of Triton X-100 0.2%. The nuclei were centrifuged at 1,000*g* for 6 min at 4 °C. The supernatant was discarded and 3 ml of sucrose buffer (1 M sucrose, 10 mM Tris-HCl pH 8.0 and 3 mM magnesium acetate with proteinase inhibitor) was carefully added from the side of the tube. The nuclei were centrifuged at 2,500*g* for 6 min at 4 °C and the supernatant was discarded. The nuclei were, thus, ready for the PLAC-seq experiment. PLAC-seq libraries were prepared as described in a previous study^17^ with minor modifications; the detailed protocol is available through the 4DN portal (https://data.4dnucleome.org/protocols/4ba366ad-b261-4545-baa0-89776c3ab699/).

### CTCF ChIP-seq data generation

First, ~30 mg of fixed tissues were thawed on ice and the nucleus preparation was performed using truChIP chromatin shearing kit (Covaris) following the manufacturer’s instructions. The lysed nuclei were sheared using Covaris M220 with the following settings: power, 75 W; duty factor, 8%; cycles per burst, 200; time, 12 min; temperature, 7 °C. The sheared chromatins were cleared by centrifugation at 15,000*g* for 12 min. The supernatant was collected and incubated with M-280 sheep anti-rabbit magnetic beads (Thermo Fisher, 11203D) for 3 h at 4 °C with slow rotation for preclearing. Then, ~5% of precleared cell lysate was saved as input control after the incubation. The remaining precleared lysate was mixed with M-280 sheep anti-rabbit magnetic beads that coupled with 0.9 μg (1:100 dilution) of anti-CTCF antibody (3418, lot 3, Cell Signaling) and rotated at 4 °C for 16 h. On the next day, the beads were collected using a magnetic stand and the supernatant was discarded. The beads were then washed with RIPA buffer (10 mM Tris pH 8.0, 140 mM NaCl, 1 mM EDTA, 1% Triton X-100, 0.1% SDS and 0.1% sodium deoxycholate) three times, high-salt RIPA buffer (10 mM Tris pH 8.0, 300 mM NaCl, 1 mM EDTA, 1% Triton X-100, 0.1% SDS and 0.1% sodium deoxycholate) twice, LiCl buffer (10 mM Tris pH 8.0, 250 mM LiCl, 1 mM EDTA, 0.5% IGEPAL CA-630 and 0.1% sodium deoxycholate) once and TE buffer (10 mM Tris pH 8.0 and 0.1 mM EDTA) twice. Washed beads were treated with 10 μg of RNase A in extraction buffer (10 mM Tris pH 8.0, 350 mM NaCl, 0.1 mM EDTA and 1% SDS) for 1 h at 37 °C, followed by reverse crosslinking in the presence of 20 μg of proteinase K (Thermo Fisher, 25530049) overnight at 65 °C. After reverse crosslinking, the DNA was purified and quantified. The input control DNA was reverse-crosslinked and purified in the same way as ChIPed DNA as described above. Library preparation was performed using a QIAseq ultralow input library kit (Qiagen) starting from 10–100 ng of ChIPed DNA or input DNA. After PCR, the amplified libraries were purified with solid-phase reversible immobilization beads to extract fragments between 200 and 600 bp for sequencing.

### CTCF ChIP-seq data processing and peak calling

The FASTQ files of CTCF ChIP-seq data were mapped to mouse genome (mm10) and processed using the ENCODE uniform processing pipeline for ChIP-seq data (https://github.com/ENCODE-DCC/chip-seq-pipeline2) with default parameters.

Peaks were called using MACS2 (ref. ^73^) with regular peak calling at a *P* threshold of 0.01. Such relaxed peak sets were generated for each biological and technical replicate, as well as for the pooled replicates. Peaks from the pooled replicate set were defined as the replicated peak set if they overlapped (at least 1 bp) with the peaks from both biological and technical replicates. FIMO^74^ with default parameters was used to search for all CTCF motifs (MA0139.1 from the JASPAR^75^ database) within the replicated peaks called from CTCF ChIP-seq data. For the sake of conservation, only replicated peaks with motifs inside were considered for furthering analysis. Filtered CTCF peaks were further annotated to promoter, exon, intron, 5′ untranslated region (UTR), 3′ UTR and distal intergenic regions by R peakAnno pacakge using the TxDb.Mmusculus.UCSC.mm10.knownGene database.

### H3K4me3 PLAC-seq data processing and interaction calling

We applied the MAPS pipeline (https://github.com/HuMingLab/MAPS) to analyze H3K4me3 PLAC-seq data, as described in our previous study^25^. Briefly, we first mapped the raw FASTQ files to the reference genome mm10 and only kept the uniquely mapped reads. We then selected valid read pairs after removing PCR duplicates. The deduped, valid read pairs were used to generate the raw contact matrix (without normalization) in the .hic format using Juicer^76^ for visualization.

We then partitioned the genome into 10-kb bins and counted the number of paired-end reads in each 10-kb bin pair. Next, we defined the ‘AND’, ‘XOR’ and ‘NOT‘ sets as 10-kb bin pairs where both two ends, only one end and neither end contained replicated H3K4me3 ChIP-seq peaks (replicated peaks from ENCODE (https://www.encodeproject.org); Supplementary Table 7) from the corresponding tissue, respectively. We only kept the bin pairs in AND and XOR sets with ≥1 count for the downstream analysis of interaction calling. We assumed that the counts in the AND and XOR sets followed a positive Poisson distribution and fitted a positive Poisson regression to adjust for systematic biases from effective fragment size, G+C content, mappability, ChIP enrichment level (measured by the number of short-range (<1 kb) reads within each 10-kb bin) and one-dimensional (1D) genomic distance. Next, we defined the normalized contact frequency as the ratio between the observed contact frequency and the expected contact frequency obtained from the positive Poisson regression. Finally, we identified long-range significant chromatin interactions at 10-kb resolution in autosomal chromosomes within a 1D genomic distance range of 20 kb–1 Mb, such that these 10-kb bin pairs satisfied FDR < 1% and normalized contact frequency ≥ 2. The significant interactions in the AND set were referred to as P2P interactions while those in the XOR set were referred to as P2N interactions.

### Quality control of PLAC-seq data

The ‘feather’ part of MAPS is a preprocessing pipeline tailored to PLAC-seq and HiChIP data and it outputs a .feather.qc file that contains metrics to evaluate the quality of PLAC-seq library. Among these metrics, the most important ones are ‘trans_ratio’, ‘long_cis_ratio’ and ‘FRiP’ (fraction of reads in peaks) values. We required all replicates in our study to satisfy the following requirements.

1. trans_ratio < 40%: trans_ratio is calculated as the number of interchromosomal read pairs in the final BAM file divided by the number of uniquely mapped read pairs that were kept after PCR duplication removal.
2. long_cis_ratio > 50% : long_cis_ratio is calculated as the number of long-range (defined by the input argument to feather, default >1,000 bp) intrachromosomal read pairs in the final BAM file after removing alternative and mitochondrial DNA (mtDNA) chromosomes (if any), divided by the number of all intrachromosomal read pairs in the final BAM file after removing alternative and mtDNA chromosomes (if any).
3. FRiP > 7.5%: FRiP is calculated as the number of ‘valid’ short-range reads (the two ends of reads coming from two different strands, intrachromosomal, ≤1,000 bp) in the final BAM file after removing alternative and mtDNA chromosomes (if any) reads that overlap with the reproducible H3K4me3 ChIP-seq peaks in the same tissue, divided by the total number of valid short-range reads.

### Merging and downsampling of H3K4me3 PLAC-seq data from the two biological replicates

The PLAC-seq data from the two biological replicates were combined by summing up the read counts in 10-kb bin pairs in the AND and XOR sets. To account for the variable sequencing depths, we further downsampled the above-combined data. Specifically, for each of 19 autosomal chromosomes, we counted the total number of paired-end reads in the AND and XOR sets across 12 tissues. We identified the tissue with the minimal number of reads and randomly downsampled the paired-end reads in the AND and XOR sets in the remaining 11 tissues to match that minimal number. After such a downsampling procedure, we ensured that, for each chromosome, the total number of paired-end reads in the AND and XOR sets are the same across 12 tissues. MAPS was applied to the combined, downsampled data of each tissue as described above to identify long-range interactions.

### Normalized contact frequency calculation and interaction calling using union H3K4me3 peaks list

Because only the AND and XOR sets were taken into consideration in MAPS, a bin pair identified as a significant interaction from one tissue might not be testable in another tissue. Thus, for valid comparison across 12 tissues, we redefined the AND and XOR sets of 10-kb bin pairs in each tissue by taking the union of H3K4me3 ChIP-seq peaks from all 12 tissues and recalculated the normalized contact frequency and long-range interactions as described above.

In this paper, for comparison analysis across tissues (Fig. 2 and Extended Data Figs. 4–6), we used the normalized contact frequency and MAPS interaction identified using the union H3K4me3 ChIP-seq peak list. Otherwise, we used replicated H3K4me3 ChIP-seq peaks from the corresponding tissue for MAPS.

### Enrichment analysis of open chromatins, histone marks and CTCF-binding sites in promoter-interacting regions

For P2N interactions, we defined the 10-kb bins that did not contain H3K4me3 peaks as promoter-interacting regions and asked whether they were enriched for open chromatins, specific histone modifications or CTCF-binding sites compared to a distance-matched control (the 10-kb bin equidistant from but on the other side of H3K4me3-containing bins). Enrichment score was defined as the fold change of the proportion of promoter-interacting regions and the control regions overlapping the midpoint of the replicated ATAC-seq, histone ChIP-seq or CTCF ChIP-seq peaks from the same tissues. The replicated peaks of each histone modification and the replicated ATAC-seq peaks of each tissue were downloaded from the ENCODE data portal (https://www.encodeproject.org/) and the identifiers are summarized in Supplementary Table 7.

### Enrichment analysis of chromatin states on promoter-interacting regions

The ChromHMM annotation files of all samples used in this study were downloaded from ENCODE (http://enhancer.sdsc.edu/enhancer_export/ENCODE/chromHMM/replicated/)^8^. The promoter-interacting regions and the control regions, which were defined above, were overlapped with the reproducible autosomal chromHMM state calls from the same tissue. The enrichment score (fold change) of a given state *S* in a particular tissue was calculated as the number of base pairs of the promoter-interacting regions that overlap state *S*, divided by the number of base pairs of the control regions that overlap state *S*.

### CTCF motif orientation analysis

Orientation of the motifs inside replicated CTCF peaks was annotated by FIMO^74^ with default parameters. If more than one CTCF motifs were found in one peak region, we only kept that with the highest FIMO score. Therefore, for each single CTCF peak, a definite and unique CTCF-binding direction could be defined on the basis of the strand of the motif (forward for those containing motifs on the positive strand and reverse for those containing motifs on the negative strand). However, when locating these CTCF peaks onto the pairs of the anchors (10-kb ends) identified by MAPS interactions, one single anchor may contain multiple CTCF peaks with opposite motif orientations. We only considered the CTCF-binding direction on an anchor as forward or reverse when it contained a single CTCF peak or multiple CTCF peaks in the same orientation. Otherwise, we referred to the anchors as bound by CTCF in dual orientations (Fig. 1i and Extended Data Fig. 3j). For CTCF convergency enrichment analysis (Extended Data Fig. 3e,f), we only considered a subset of MAPS-identified interactions with both ends containing either a single CTCF motif or multiple CTCF motifs in the same direction.

### Identification of interacting cCRE–gene and cEnhancer–gene pairs

PLAC-seq interactions between promoter-distal elements and active transcription start sites (TSSs) (XOR set from the MAPS software) were used to determine all cCRE–gene interactions. BEDTools intersect (https://bedtools.readthedocs.io/en/latest/) was used to determine all chromatin-accessible peaks from the d-TAC catalog^8^ overlapping a 10-kb nonanchor bin (cCREs). cCREs were assigned to protein-coding genes with an H3K4me3-marked TSS (Gencode vM4) in the 10-kb anchor bin for each interaction. This generated a catalog of 91,451 interacting cCRE–gene pairs. All integrated data were from the same tissue type and at the same developmental stage. To determine cEnhancers, these pairs were filtered for having cCRE H3K27ac signal that correlated with interacting gene expression. HTSeq software (https://htseq.readthedocs.io/en/master/) was used to calculate all H3K27ac counts within a 2-kb window from the center of each interacting cCRE for each tissue and developmental stage. H3K27ac counts were normalized for total counts and values were quantile-normalized. SCCs for normalized H3K27ac counts at cCREs with the interacting gene’s FPKM (fragments per kilobase of transcript per million mapped reads) value^24^ for each interacting cCRE–gene pair were calculated. Biological replicate datasets for each sample were kept separate unless H3K27ac ChIP-seq and mRNA-seq were generated from different samples, in which case duplicates were combined and averaged. This resulted in 22 data points for SCC calculation (Supplementary Table 3). Those with a positive SCC and FDR < 0.05 were included in the list of cEnhancer–gene pairs. FDR values were calculated from *P* values using the Benjamini–Hochberg procedure.

### Comparison of the closest promoter cCRE assignment with interacting cCRE promoter pairs

Correlations between H3K27ac at cCREs and the expression of the assigned target gene were compared between interacting cCRE–gene pairs (*n* = 91,451, described above) and cCRE–closest promoter pairs (*n* = 115,711). For cCRE–closest promoter pairs, only cCREs that were between 20 kb and 1 Mb from the nearest promoter were considered to match the distances between interacting cCREs and target genes. Gencode vM4 (GRCm38.p3) was used for protein-coding TSS coordinates. SCCs were calculated for each group and plotted as a function of the distance between cCREs and TSSs. For genes with alternative TSSs, the distance to the TSS with the highest H3K27ac signal was considered. A spline fit was illustrated using R (smooth.spline function, degrees of freedom = 2). A two-sample *t*-test, two-sided for each 200-kb interval, was performed.

### Clustering analysis of cEnhancer–gene pairs by tissue-specific and stage-specific H3K27ac

Two groups of cEnhancer–gene pairs were created: (1) pairs with an MAPS-called interaction at E12.5 in one or more tissue type (*n* = 9,370) and (2) pairs with an MAPS-called interaction in one or more stages of developing FB (*n* = 11,893). The *k*-means clustering was performed on log_2_-normalized H3K27ac read counts within 2 kb of the center of the ATAC peak of cEnhancers. We chose to use four main clusters for E12.5 (Fig. 3e) and five main clusters for FB (Extended Data Fig. 8b) cEnhancer–gene pairs because adding additional clusters did not reveal new patterns of cEnhancer or gene activity between samples. Subclustering was performed for clusters that had high dynamic ranges for signal intensities. Heat maps were generated using Java TreeView software. Additional sample-specific chromatin features were included. For ChIP-seq data, counts were considered within 2 kb of the ATAC peak center for cEnhancers and 2 kb of the TSS of target genes. Values were quantile-normalized and log_2_-transformed.

### Enhancer validation using VISTA elements and sequence conservation

Validation rates for all cCREs, interacting cCREs, predicted enhancers from Gorkin et al.^8^ and cEnhancers were calculated by determining their ratio of overlapping positive to overlapping negative VISTA elements for in vivo enhancer activity in any tissue. The numbers of referenced VISTA elements were 1,618 positive and 1,481 negative, which were converted from mm9 to mm10 coordinates using the UCSC LiftOver tool (https://genome.ucsc.edu/cgi-bin/hgLiftOver) with default settings. Genes assigned to positive VISTA enhancers were determined for each E12.5 tissue assayed by PLAC-seq. Expression distribution and GO analyses were performed on genes that interacted with a VISTA enhancer element that had positive in vivo activity in the same tissue where a PLAC-seq interaction was observed. Sequence conservation for each element was scored by taking the average phastCons score for each base pair. The phastConsElements60way and phastConsElements60wayPlacental conserved elements were downloaded from the UCSC genome browser. Shuffled control regions were generated using BEDTools shuffle with the coordinates of all cCREs as input. H3K27ac-matched regions for cEnhancers were selected from the set of all cCREs after excluding cEnhancers. The distributions of log_2_-normalized H3K27ac read counts within 2 kb of the center of the ATAC peak were divided into 100 equal-frequency bins for all cCREs. The number of cEnhancers from each bin was counted and the same number of randomly selected non-cEnhancer cCREs were selected from the corresponding bin.

### GO and TF motif enrichment analysis

GO analysis was performed on gene lists using the HOMER findGO.pl function with default parameters. The top enriched GO biological process terms were reported. TF motif enrichment was performed using the HOMER findMotifsGenome.pl function ±200 bp from the cCRE center. Top enriched de novo and known TF motifs were reported for TF gene expression patterns that correlated with TF motif enrichment across tissues.

### GWAS enrichment of cEnhancers and SCZ SNP fine mapping

ATAC peak regions of E12.5 interacting cEnhancers were converted from mm10 to hg19 coordinates using liftover with setting ‘-minMatch 0.5’. Regions that did not map back to their original mm10 coordinates were discarded. Using summary statistics for a panel of traits and diseases^77^, we performed LDSC regression on cEnhancers from E12.5 clusters K1, K2 and K3 with all cEnhancers as a local background control set. SCZ 99% credible set SNPs^67^ were leveraged to find cEnhancers overlapping with candidate causal genetic variants.

### Reporting summary

Further information on research design is available in the Nature Portfolio Reporting Summary linked to this article.

## Online content

Any methods, additional references, Nature Portfolio reporting summaries, source data, extended data, supplementary information, acknowledgements, peer review information; details of author contributions and competing interests; and statements of data and code availability are available at 10.1038/s41594-024-01431-2.

## Supplementary information

Supplementary Information Supplementary Figs. 1 and 2 and Methods.

Reporting Summary

Supplementary Tables 1–7 Supplementary Tables 1–7.

Supplementary Data 1 The list of MAPS interactions using the union H3K4me3 ChIP-seq peaks from all 12 tissues, related to Fig. 2 and Extended Data Figs. 4–6.

## Source data

Source Data Fig. 2 Source data for the heat map.

Source Data Extended Data Fig. 6 Source data for the heat map.

## Data Availability

The H3K4me3 PLAC-seq and CTCF ChIP-seq datasets generated in this study were deposited to the Gene Expression Omnibus with accession number GSE200114. The H3K4me3 PLAC-seq datasets are also available from the 4DN data portal with accession codes 4DNESG7Q6HPT, 4DNES8IEZPCJ, 4DNES8DEXNEY, 4DNESS2HA1AN, 4DNES2VIMAYW, 4DNESA85FV7T, 4DNESTLK5GLR, 4DNESBUE56SA, 4DNESV96LIGH, 4DNESO2R26BF, 4DNESR5QMPXM and 4DNESGAQJIF8. The processed files of ChIP-seq, RNA-seq and ATAC-seq data for the mouse embryonic tissues were downloaded from the ENCODE portal (https://www.encodeproject.org/; identifiers in Supplementary Table 7). The Gencode vM4 annotation was downloaded from ENCODE (https://www.encodeproject.org/data-standards/reference-sequences/). The mouse E10.5–E13.5 LM and MB Capture-C datasets were downloaded from the Gene Expression Omnibus under accession number GSE84795. Source data are provided with this paper.

## Code availability

The analysis of PLAC-seq and ChIP-seq data in this study was performed using publicly available software described in Methods.
