# Supplementary material for: Integrative analysis of the 3D genome and epigenome in mouse embryonic tissues
Source: Nat Struct Mol Biol. 2024 Dec 16;32(3):479–90. doi: 10.1038/s41594-024-01431-2 (PMC11919700; doi:10.1038/s41594-024-01431-2)
Supplement: Supplementary file 1 — Supplementary Figs. 1 and 2 and Methods. [file 41594_2024_1431_MOESM1_ESM.pdf]

nature structural & molecular biology

Supplementary information https://doi.org/10.1038/s41594-024-01431-2

Integrative analysis of the 3D genome and epigenome in mouse embryonic tissues

In the format provided by the authors and unedited

Supplementary information

Supplementary Figure

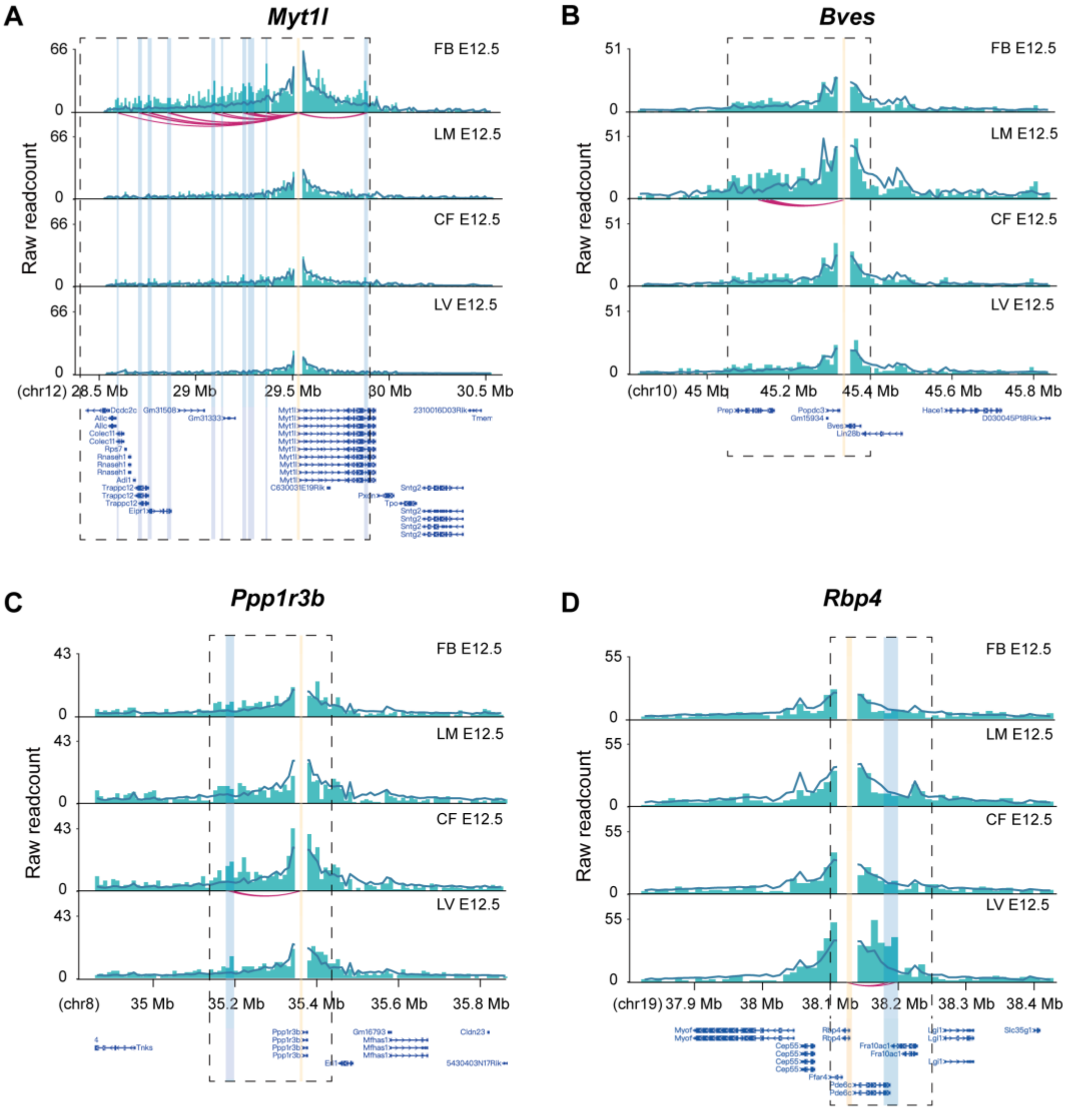

Supplementary Figure 1. Examples of PLAC-seq interaction visualization

Virtual 4C plots of H3K4me3 PLAC-seq signal anchored at TSS regions around *Myt1l* (**A**), *Bves* (**B**), *Ppp1r3b* (**C**), *Rbpv* (**D**) in forebrain (FB), limb (LM), craniofacial prominence (CF) and liver (LV) at embryonic day 12.5 (E12.5) under 10-kb resolution. The bars represent the raw readcount in H3K4me3 PLAC-seq on each bin and their expected counts (both distance and H3K4me3 signal strength on the bin are considered) are showed by the blue line. Similar to **Fig. 2d**, the bins containing H3K4me3 peaks (around TSS) are highlighted by yellow boxes. Bins located within 20-kb of the anchor bin are not considered in MAPS interaction calling and thus filled with blank. Bins with raw read count larger than two folds of the expected counts and FDR < 1% are highlighted since MAPS identified such bins as forming interaction to the anchor region, as shown by the purple arc.

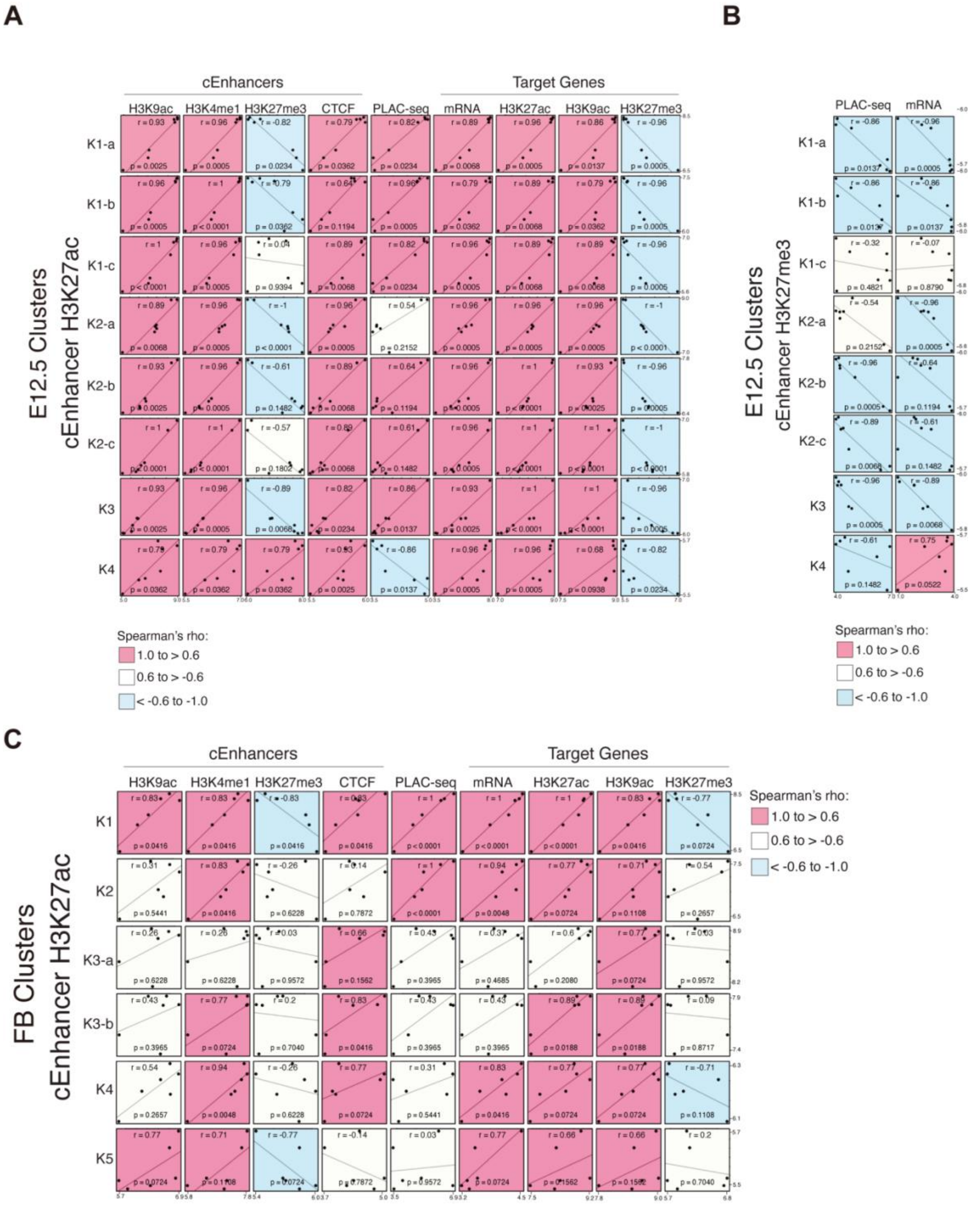

Supplementary Figure 2. Correlation between cEnhancer H3K27ac, H3K27me3 with contact frequency and other histone modifications.

A. Scatterplots displaying correlations between cEnhancer H3K27ac and each column across E12.5 clusters from **Fig. 3d**.

B. Scatterplots displaying correlations between cEnhancer H3K27me3 and PLAC-seq and mRNA across E12.5 clusters from **Fig. 3d**.

C. Scatterplots displaying correlations between cEnhancer H3K27ac and each column across FB clusters from **Extended Data Fig. 8b.**

Supplementary Method

Reproducibility analysis of MAPS-identified interactions from two biological replicates.

After acquiring the MAPS-identified long-range chromatin interactions from each biological replicate, two approaches were used to evaluate their reproducibility: (1) we calculated the overlap between MAPS-identified interactions from two replicates, (2) we calculated the Pearson correlation coefficients of the normalized contact frequency for the reproducible interactions from two replicates.

Gene and TSS Annotation

We downloaded Gencode vM4 annotation (https://www.encodeproject.org/data-standards/reference-sequences/) to define transcript TSS for each protein coding gene. To count number of genes involved in or in PLAC-seq interactions, we only considered whether the TSS of the gene is laid in either of the 10 Kb ends of the PLAC-seq interactions.

Comparison of MAPS-identified interactions with Capture-C interactions

The Capture-C interactions from forelimb and hindlimb at E10.5, E11.5 and E13.5, and midbrain at E12.5 were downloaded from Gene Expression Omnibus (GEO) with accession number GSE84795, using the 95th or the 99th percentile as the cut-off value[1]. The mm9 coordinates used in the original paper were converted to mm10 using UCSC LiftOver tool (https://genome.ucsc.edu/cgi-bin/hgLiftOver). Since MAPS only detected interactions that have at least one end overlapping H3K4me3 peaks, we further filtered Capture-C interactions for fair comparison. Specifically, a Capture-C interaction was considered as testable only when it satisfies the following two criteria: (1) the interaction was with 1D genomic distance between 20 Kb to 1 Mb in autosomal chromosomes and (2) the interaction has at least one end overlapping the 10 Kb bins containing reproducible H3K4me3 peaks identified in the corresponding tissue (LM E12.5 for forelimb and hindlimb Capture-C data and MB E12.5 for midbrain E10.5 Capture-C data).

Since PLAC-seq and Capture-C were not performed on exactly the same tissues, the MAPS-identified interactions of MB E12.5 were compared with Capture-C interactions from midbrain E10.5, and the MAPS-identified interactions of LM E12.5 were compared with Capture-C interactions from forelimb and hindlimb. A testable Capture-C interaction was considered as being recapitulated by PLAC-seq when its both ends overlapped the two ends (at least 1 bp) of a PLAC-seq interaction identified from the corresponding tissues. As a control set, a list of distance-matched pseudo-interactions was generated by linking the H3K4me3-containing 10 Kb bin to the 10 Kb bin equidistant from but on the other side of H3K4me3-containing bins. For tissue-specific comparison, interactions identified from all 6 forelimb and hindlimb tissues were combined and deduplicated as the pooled limb (LM-pooled) interaction list and all Capture-C interactions were further classified into two categories: interactions that can be identified in both tissue types (MB E10.5 and pooled-LM) were referred to as 'shared interactions,' while interactions that can only be detected in one type (MB E10.5 or pooled-LM) were referred to as 'tissue-specific interactions.

Clustering of PLAC-seq data across 12 tissues

Clustering analysis including principal component analysis (PCA) and hierarchical clustering analysis (HCA) was performed on the normalized contact frequency of 10 Kb bin pairs which were called as significant interactions in at least 1 of the 12 tissues using union H3K4me3 peak list. Bin pairs with zero variance of normalized contact frequency across 12 tissues were discarded. Finally, a total of 195009 bin pairs were used for clustering analysis. Based on the normalized contact frequency matrix of these 195009 bin pairs, PCA was performed by R function “prcomp” and HCA was performed by the “hclust” function in R using Ward's minimum variance method (using ‘ward.D’ argument).

Clustering of H3K27ac ChIP-seq data across 12 tissues

Hierarchical clustering of H3K27ac histone modification ChIP-seq was performed using the “hclust” function in R as previously described[2]. First, replicated H3K27ac ChIP-seq peaks from all 12 tissues were pooled together, merging by bedtools merge (v2.29.2)[3] so that the

same peak list was used across all 12 tissues for comparison. Average fold enrichments on these merged peaks were re-calculated by bigWigAverageOverBed (https://github.com/ENCODE-DCC/kentUtils/blob/master/bin/linux.x86_64/bigWigAverageOverBed), using pooling signal (fold change over control) bigwig files of each tissue as input. Finally, quantile normalization was applied and the normalized scores were used for hierarchical clustering. The replicated peaks of H3K27ac histone modification as well as the signal bigwig files were downloaded from ENCODE data portal (https://www.encodeproject.org/) and the identifiers are summarized in **Supplementary Table 7**.

Heatmap for tissue- or stage-specific interaction features.

We determined tissue or stage-specific XOR MAPS interactions as those having significance in only the indicated E12.5 tissues (**Fig. 2b**) or FB developmental stage (**Extended Data Fig. 6a**). For valid comparisons across samples, we used normalized contact frequencies from the union set anchor interactions. Bedtools[3] pairToBed function was used to find H3K4me3 marked promoters in the anchor bin and H3K27ac peaks in the non-anchor bin. Heatmap displays the percentage of an individual tissue FPKM of the total sum of FPKM for all tissues for each gene. H3K27ac signal was calculated by counts within peaks overlapping with the non-anchor interacting bins. H3K27ac counts were corrected for total mapped reads and quantile normalized. Heatmap displays the percentage of an individual tissue H3K27ac counts of the total sum of H3K27ac counts for all tissues for each non-anchor bin.

Correlation analysis between fold-change of gene expression and that of number of MAPS-identified significant chromatin interactions

We first selected 9,939 protein-coding genes which are expressed in at least one of 12 tissues (defined by FPKM>1), and have transcription start site (TSS) overlaps with H3K4me3 ChIP-seq peak in at least one of 12 tissues. Next, we converted the FPKM value to Log2(FPKM+1), and applied R function “normalize.quantile” in the “preprocessCore” library to perform quantile normalization across all 12 tissues. The log2 transformed and quantile normalized gene expression data for these 9,939 genes were used for the downstream

analysis. In addition, we counted the number of the number of MAPS-identified significant chromatin interactions in each tissue. For any two tissues, we further made the scatter plot between the change of chromatin interaction and the change of gene expression, and reported the Pearson correlation coefficients and the corresponding P-values.

Comparison of cEnhancer/cEnhancer-gene ortholog pairs with human cCREs/cCRE-gene from human fetal brain dataset.

cEnhancers were converted from mm10 to hg38 coordinates using liftover with settings -minMatch 0.5. Regions that did not map back to their original mm10 coordinates were discarded. To compare cEnhancer orthologs with human cCREs, we defined human cCREs as the 210,392 pooled ATAC-seq peaks identified from four cell types isolated from the human cortex in the Song et al. study[4], or the 656,891 consensus ATAC peaks identified from the 22 cell clusters in the Trevino et al. study[5]. Regions of equal size to the cEnhancer ortholog, equidistant distance from but on the other side of the target gene, were used as control. To compare cEnhancer-gene ortholog pairs with human cCRE-gene pairs, we considered only pairs with a human-recaptured cEnhancer ortholog. Pseudo-pairs linking the target genes with the control regions were used as controls. We considered a cEnhancer-gene ortholog pair (or a control-gene pair) to be supported by the human dataset when it overlapped with both ends of a PLAC-seq interaction in the Song et al. study or when the cEnhancer ortholog was assigned to the same target gene, as in the Trevino et al. study.

Supplementary Method References

1. Andrey, G. *et al.* Characterization of hundreds of regulatory landscapes in developing limbs reveals two regimes of chromatin folding. *Genome Research* **27**, 223-233 (2017).
2. Gorkin, D.U. *et al.* An atlas of dynamic chromatin landscapes in mouse fetal development. *Nature* **583**, 744-751 (2020).
3. Quinlan, A.R. & Hall, I.M. BEDTools: A flexible suite of utilities for comparing genomic features. *Bioinformatics* **26**, 841-842 (2010).
4. Song, M. *et al.* Cell-type-specific 3D epigenomes in the developing human cortex. *Nature* **587**, 644-649 (2020).
5. Trevino, A.E. *et al.* Chromatin and gene-regulatory dynamics of the developing human cerebral cortex at single-cell resolution. *Cell* **184**, 5053-5069 e23 (2021).
