# Supplementary material for: Integrative analysis of the 3D genome and epigenome in mouse embryonic tissues
Source: Nat Struct Mol Biol. 2024 Dec 16;32(3):479–90. doi: 10.1038/s41594-024-01431-2 (PMC11919700; doi:10.1038/s41594-024-01431-2)
Supplement: Supplementary file 2 — Reporting Summary [file 41594_2024_1431_MOESM2_ESM.pdf]

nature portfolio

Corresponding author(s): Miao Yu, Ming Hu, and Bing Ren

Last updated by author(s): Sep 3, 2024

Reporting Summary

Nature Portfolio wishes to improve the reproducibility of the work that we publish. This form provides structure for consistency and transparency in reporting. For further information on Nature Portfolio policies, see our Editorial Policies and the Editorial Policy Checklist.

Statistics

For all statistical analyses, confirm that the following items are present in the figure legend, table legend, main text, or Methods section.

n/a	Confirmed	
☐	☒	The exact sample size (*n*) for each experimental group/condition, given as a discrete number and unit of measurement
☐	☒	A statement on whether measurements were taken from distinct samples or whether the same sample was measured repeatedly
☐	☒	The statistical test(s) used AND whether they are one- or two-sided<br>*Only common tests should be described solely by name; describe more complex techniques in the Methods section.*
☐	☒	A description of all covariates tested
☐	☒	A description of any assumptions or corrections, such as tests of normality and adjustment for multiple comparisons
☐	☒	A full description of the statistical parameters including central tendency (e.g. means) or other basic estimates (e.g. regression coefficient) AND variation (e.g. standard deviation) or associated estimates of uncertainty (e.g. confidence intervals)
☐	☒	For null hypothesis testing, the test statistic (e.g. *F*, *t*, *r*) with confidence intervals, effect sizes, degrees of freedom and *P* value noted<br>*Give P values as exact values whenever suitable.*
☒	☐	For Bayesian analysis, information on the choice of priors and Markov chain Monte Carlo settings
☐	☒	For hierarchical and complex designs, identification of the appropriate level for tests and full reporting of outcomes
☐	☒	Estimates of effect sizes (e.g. Cohen's *d*, Pearson's *r*), indicating how they were calculated

Our web collection on statistics for biologists contains articles on many of the points above.

Software and code

Policy information about availability of computer code

Data collection	No software was used for data collection.
Data analysis	MAPS v1.1.0 (https://github.com/HuMingLab/MAPS), UCSC LiftOver tool v377 (https://genome.ucsc.edu/cgi-bin/hgLiftOver), FIMO v4.9.1 (https://meme-suite.org/meme/doc/fimo.html), bedtools v2.29.2 (https://bedtools.readthedocs.io/en/latest/), Homer v4.11 (http://homer.ucsd.edu/homer/), MACS2 v2.2.4 (https://github.com/macs3-project/MACS), ENCODE ChIP-seq pipeline (https://github.com/ENCODE-DCC/chip-seq-pipeline2), HTSeq software 0.12.4 (https://htseq.readthedocs.io/en/master/), Java TreeView software 3.0

For manuscripts utilizing custom algorithms or software that are central to the research but not yet described in published literature, software must be made available to editors and reviewers. We strongly encourage code deposition in a community repository (e.g. GitHub). See the Nature Portfolio guidelines for submitting code & software for further information.

Data

Policy information about availability of data

All manuscripts must include a data availability statement. This statement should provide the following information, where applicable:

- Accession codes, unique identifiers, or web links for publicly available datasets
- A description of any restrictions on data availability
- For clinical datasets or third party data, please ensure that the statement adheres to our policy

The H3K4me3 PLAC-seq and CTCF ChIP-seq datasets generated in this study have been deposited to the Gene Expression Omnibus (GEO) with accession number

GSE200114. The H3K4me3 PLAC-seq datasets were also available from 4DN Data portal (https://data.4dnucleome.org, with accession code 4DNESG7Q6HPT, 4DNES8IEZPCJ, 4DNES8DEXNEY, 4DNESS2HA1AN, 4DNES2VIMAYW, 4DNESA85FV7T, 4DNESTLK5GLR, 4DNESBUE56SA, 4DNESV96LIGH, 4DNESO2R26BF, 4DNESR5QMPX and 4DNESGAQJIF8). The processed files of ChIP-seq, RNA-seq and ATAC-seq data for the mouse embryonic tissues were downloaded from the ENCODE portal (https://www.encodeproject.org/, the identifiers provided in Supplementary Table 4). The Gencode vM4 annotation was downloaded from https://www.encodeproject.org/data-standards/reference-sequences/. The mouse E10.5-E13.5 limb and midbrain Capture-C datasets were downloaded from https://www.ncbi.nlm.nih.gov/geo/query/acc.cgi?acc=GSE84795. Source data are provided with this paper.

Human research participants

Policy information about studies involving human research participants and Sex and Gender in Research.

Reporting on sex and gender	N/A
Population characteristics	N/A
Recruitment	N/A
Ethics oversight	N/A

Note that full information on the approval of the study protocol must also be provided in the manuscript.

Field-specific reporting

Please select the one below that is the best fit for your research. If you are not sure, read the appropriate sections before making your selection.

☒ Life sciences ☐ Behavioural & social sciences ☐ Ecological, evolutionary & environmental sciences

For a reference copy of the document with all sections, see nature.com/documents/nr-reporting-summary-flat.pdf

Life sciences study design

All studies must disclose on these points even when the disclosure is negative.

Sample size	12 mouse fetal tissues were chosen in this study to provide sufficient data points to predict gene-cEnhancer pairs.
Data exclusions	No data were excluded.
Replication	Two biological replicates were used for PLAC-seq experiments with high reproducible contact frequencies (Pearson correlation coefficients 0.77 - 0.80). Two biological replicates/technical replicates were used for CTCF ChIP-seq experiments with high reproducibility of peak region signals as determined by Pearson correlation coefficients 0.8 and above.
Randomization	No randomization. The study were performed using 7 different mouse fetal tissues from certain time points.
Blinding	Blinding is not relevant to this study since no subjective measurements were taken during data collection/analysis.

Reporting for specific materials, systems and methods

We require information from authors about some types of materials, experimental systems and methods used in many studies. Here, indicate whether each material, system or method listed is relevant to your study. If you are not sure if a list item applies to your research, read the appropriate section before selecting a response.

Materials & experimental systems

n/a	Involved in the study	
☐	☒	Antibodies
☒	☐	Eukaryotic cell lines
☒	☐	Palaeontology and archaeology
☐	☒	Animals and other organisms
☒	☐	Clinical data
☒	☐	Dual use research of concern

Methods

n/a	Involved in the study	
☐	☒	ChIP-seq
☒	☐	Flow cytometry
☒	☐	MRI-based neuroimaging

Antibodies

Antibodies used	H3K4me3 antibody for PLAC-seq (cat. no. 04-745, clone MC315, Millipore,); CTCF antibody for ChIP-seq (cat. no. 3418, clone D31H2, Cell Signaling)
Validation	Anti-trimethyl-Histone H3 (Lys4) Antibody, clone MC315 is a rabbit monoclonal antibody for detection of human and mouse H3K4me3 and has been validated by ENCODE. The validation results can be found at: https://www.encodeproject.org/antibodies/ENCAB610CEF/.<br>The CTCF antibody from Cell Signaling (3418) is a rabbit monoclonal antibody and its species reactivity includes human, mouse, rat and monkey. It has been validated by ENCODE and the results can be found at: https://www.encodeproject.org/antibodies/ENCAB498PCM/.

Animals and other research organisms

Policy information about studies involving animals; ARRIVE guidelines recommended for reporting animal research, and Sex and Gender in Research

Laboratory animals	Tissue collection was performed using female C57BL/6N Mus musculus animals. Embryonic mice of E12.5, E13.5, E14.5, E15.5, E16,5 and p0 from pregnent female mice were collected.
Wild animals	This study did not involve wild animals.
Reporting on sex	Biological sex is not visually obvious for the the developmental stages in our study and was not assessed.
Field-collected samples	No field samples were collected.
Ethics oversight	All animal work was reviewed and approved by the Lawrence Berkeley National Laboratory Animal Welfare and Research Committee.

Note that full information on the approval of the study protocol must also be provided in the manuscript.

ChIP-seq

Data deposition

☒ Confirm that both raw and final processed data have been deposited in a public database such as GEO.

☒ Confirm that you have deposited or provided access to graph files (e.g. BED files) for the called peaks.

Data access links<br>*May remain private before publication.*	https://www.ncbi.nlm.nih.gov/geo/query/acc.cgi?acc=GSE200114.
Files in database submission	For H3K4me3 PLAC-seq data: raw fastq files, .hic files, and .bedpe files for the called long-range interactions.<br>For CTCF ChIP-seq data: raw fastq files, bigwig file representing the signal track for fold change over control. Replicated peak files in narrowPeak format.
Genome browser session<br>(e.g. UCSC)	not applicable

Methodology

Replicates	Two biological replicates were used for PLAC-seq experiments with high reproducible contact frequencies (Pearson correlation coefficients 0.77 - 0.80). Two biological replicates/technical replicates were used for CTCF ChIP-seq experiments with high reproducibility of peak region signals as determined by Pearson correlation coefficients ~ 0.8 and above.
Sequencing depth	Each PLAC-seq sample was sequenced to a median sequencing depth of 206 million reads and the detailed numbers can be found in Supplementary Table 1.
Antibodies	H3K4me3 antibоday (cat. no. 04-745, clone MC315, Millipore) for PLAC-seq assay; CTCF antibody (cat. no. 3418, clone D31H2, Cell Signaling) for CTCF ChIP-seq assay
Peak calling parameters	Peaks were called using MACS2 with regular peak calling at P threshold of 0.01. Such relaxed peak sets were generated for each biological/technical replicate, and also for the pooled replicates. Peaks from the pooled replicate set were defined as the replicated peak set if they overlapped (at least 1 bp) the peaks from both biological/technical replicates.
Data quality	Between 30,000 and 60,000 CTCF peaks were reproduced between replicates. The majority of peaks contained a CTCF motif > 80%.
Software	The fastq files of CTCF ChIP-seq data were mapped to mouse genome (mm10) and processed using the ENCODE uniform processing pipeline for ChIP-seq data (https://github.com/ENCODE-DCC/chip-seq-pipeline) with default parameters.
